# Design and Synthesis of Helical *N*-Terminal l-Prolyl Oligopeptides Possessing Hydrocarbon Stapling

**DOI:** 10.3390/molecules25204667

**Published:** 2020-10-13

**Authors:** Atsushi Ueda, Mei Higuchi, Kazuki Sato, Tomohiro Umeno, Masakazu Tanaka

**Affiliations:** Graduate School of Biomedical Sciences, Nagasaki University, 1-14 Bunkyo-machi, Nagasaki 852-8521, Japan; m.higuchi005@gmail.com (M.H.); bb55619010@ms.nagasaki-u.ac.jp (K.S.); umeno@ac.shoyaku.ac.jp (T.U.)

**Keywords:** peptide, helix, hydrocarbon stapling, ring-closing metathesis, organocatalyst, l-proline, Michael addition

## Abstract

We designed and synthesized helical short oligopeptides with an l-proline on the N-terminus and hydrocarbon stapling on the side chain. Side-chain stapling is a frequently used method for the development of biologically active peptides. Side-chain stapling can stabilize the secondary structures of peptides, and, therefore, stapled peptides may be applicable to peptide-based organocatalysts. Olefin-tethered *cis*-4-hydroxy-l-proline **1** and l-serine **2** and **8**, and (*R*)-α-allyl-proline **18** were used as cross-linking motifs and incorporated into helical peptide sequences. The *Z*- and *E*-selectivities were observed for the ring-closing metathesis reactions of peptides **3** and **11** (*i*,*i*+1 series), respectively, while no *E*/*Z*-selectivity was observed for that of **19** (*i*,*i*+3 series). The stapled peptide **B’** catalyzed the Michael addition reaction of 1-methylindole to α,β-unsaturated aldehyde, which was seven times faster than that of unstapled peptide **B**. Furthermore, the high catalytic activity was retained even at lower catalyst loadings (5 mol %) and lower temperatures (0 °C). The circular dichroism spectra of stapled peptide **B’** showed a right-handed helix with a higher intensity than that of unstapled peptide **B**. These results indicate that the introduction of side-chain stapling is beneficial for enhancing the catalytic activity of short oligopeptide catalysts.

## 1. Introduction

Hydrocarbon stapling is one of the most commonly used methods to stabilize the secondary structure of peptides as a way to provide enhanced functionality [1,2,3]. This powerful tool is especially important for short oligopeptides due to their flexible secondary structure. Grubbs et al. reported the synthesis of 3_10_-helical heptapeptides stabilized by hydrocarbon stapling at the *i*,*i*+4 positions using ring-closing metathesis [4,5]. In 2000, Verdine’s group reported all-hydrocarbon stapling using α-olefin-tethered alanine at *i*,*i*+4 as well as at *i*,*i*+7 positions, and the introduction of these staples highly induced α-helicities as well as the metabolic stabilities of the peptides [6]. Nowadays, the all-hydrocarbon staplings at the *i*,*i*+4 and *i*,*i*+7 positions are widely used in the medicinal chemistry of peptides [7,8,9].

**Figure 1:**
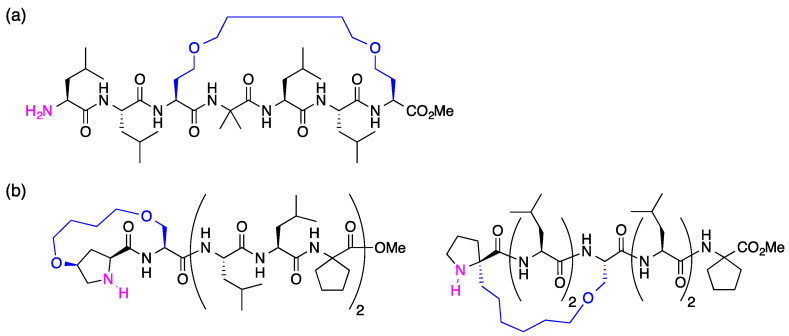
Hydrocarbon-stapled peptides used in the organocatalytic reaction. (**a**) Helical peptide-based primary amino catalyst with stapling at *i*,*i*+4 and (**b**) helical peptide-based secondary amino catalysts with stapling at *i*,*i*+1 and *i*,*i*+3 (This work).

Short peptides are also attractive compounds in the field of organocatalysis [10,11,12,13]. These peptide catalysts can be categorized into several classes based on their secondary structure, those including β-turn [14,15], helix [16,17,18,19,20], turn-helix types [21,22,23], and so forth. Therefore, hydrocarbon stapling can be an effective tool for the development of peptide-based organocatalysts by controlling their secondary structure. However, there are few examples of stapled peptide-catalyzed asymmetric organocatalytic reactions. Demizu and co-workers reported an enantioselective Juliá–Colonna epoxidation of chalcone catalyzed by a helical peptide-based primary amino catalyst possessing a crosslink between two l-homoserines at the *i*,*i*+4 positions (Figure 1) [24,25]. Likewise, secondary amino catalysts are powerful catalysts with a broad range of applicable reactions [26,27]. Moreover, the peptide hydrocarbon staplings at the *i*,*i*+1 and *i*,*i*+3 [28,29] positions are rarely examined compared to *i*,*i*+4 series but have potential as constrained cyclic peptides for organocatalysis, material science, drug discovery, and so forth [30]. From this point of view, the introduction of allyl tethered *cis*-4-hydroxy-l-proline or (*R*)-α-allyl-proline can be suitable for this purpose. Secondary structures, as well as helical screw directions, can be controlled by introducing 1-aminocycloalkane-1-carboxylic acid in homopeptides [31,32,33,34,35,36,37,38,39,40] and heteropeptides [41,42,43], and these constrained peptides catalyze asymmetric 1,4-addition reactions [44,45,46]. Therefore, poly l-leucine-incorporating 1-aminocyclopentane-1-carboxylic acid was used as an α-helix-inducing motif. The stapling efficiency was evaluated by comparing the catalytic activities of stapled and unstapled peptides in Friedel–Crafts type 1,4-addition reactions [47,48]. Herein, we report the synthesis of helical *N*-terminal prolyl oligopeptides with hydrocarbon stapling at *i*,*i*+1 as well as *i*,*i*+3 positions and the enhancements of their catalytic activity for the Michael addition of 1-methylindole to α,β-unsaturated aldehyde.

## 2. Results and Discussion

**Scheme 1:**
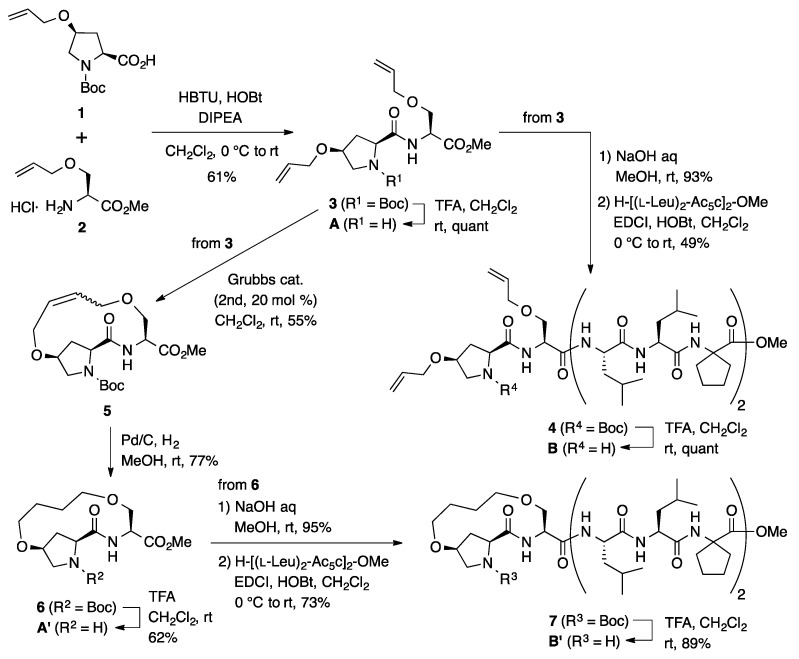
Synthesis of unstapled peptides **A** and **B** and stapled peptides **A’** and **B’** (tethering at *i*, *i*+1 positions with a 13-membered ring).

The synthesis of the unstapled peptides **A** and **B** and stapled peptides of the *i*,*i*+1 series **A’** and **B’** began from allyl tethered *cis*-4-hydroxy-l-proline **1** [49,50] and l-serine **2**, as illustrated in Scheme 1. The coupling of **1** and **2** produced dipeptide **3**, which was successfully introduced to a helix-inducing motif, H-(l-Leu-l-Leu-Ac_5_c)_2_-OMe [41,44]. The deprotection of the Boc-protecting groups of **3** and **4** produced *N*-terminal free peptides **A** and **B** in quantitative yields. On the other hand, the ring-closing metathesis of dipeptide **3** was performed with 20 mol % of the second-generation Grubbs catalyst to give *i*,*i*+1-stapled dipeptide **5**. In this reaction, the *Z*-configured product was obtained as a major product (*E*/*Z* = 1.0:5.6), possibly due to the medium ring size of **5** (13-membered ring) along with the rigid 4-hydroxyproline part. The hydrogenation of **5** provided dipeptide **6**, which was coupled with H-(l-Leu-l-Leu-Ac_5_c)_2_-OMe to afford stapled octapeptide **7**. The *N*-Terminal free peptides **A’** and **B’** were obtained by the Boc-deprotection of **6** and **7**, respectively. It should be noted that neither the ring-closing metathesis of octapeptide **4** nor that of the *trans*-4-hydroxy-l-proline derivatives of **3** produced the desired cyclization products. This poor reactivity may be caused by a ring strain of the 13-membered ring product, which resulted in a preference for the *Z*-configured isomer of **5**.

**Scheme 2:**
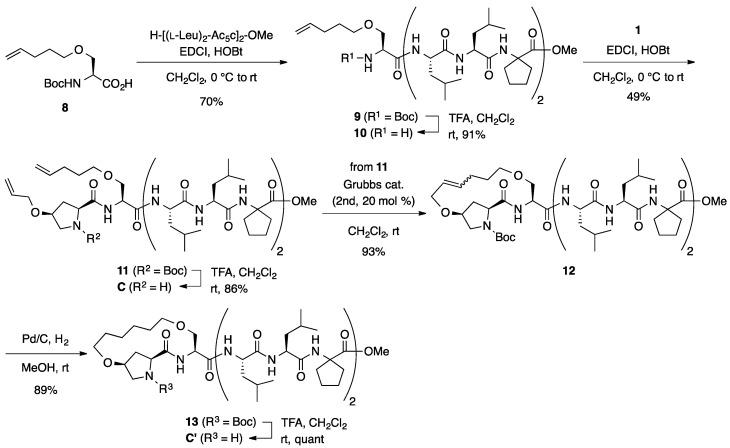
Synthesis of unstapled peptide **C** and stapled peptide **C’** (tethering at *i*, *i*+1 positions with a 15-membered ring).

Stapled peptide **C’** possessing tethered side chains at the *i*,*i*+1 positions with a 15-membered macrocyclic ring was also synthesized from 4-pentenyl tethered l-Ser **8** [51] by a similar manner as described in Scheme 1 (Scheme 2). In contrast to the reaction of **4** to **7**, unstapled peptide **11** underwent the ring-closing metathesis reaction smoothly to provide stapled peptide **12** in a 93% yield with a preference for *E*-isomer over *Z*-isomer (*E*/*Z* = 5.5:1.0). This smooth reaction implies the released macrocyclic ring strain of the product, which resulted in a preference for thermodynamically favored *E*-olefin isomer.

**Scheme 3:**
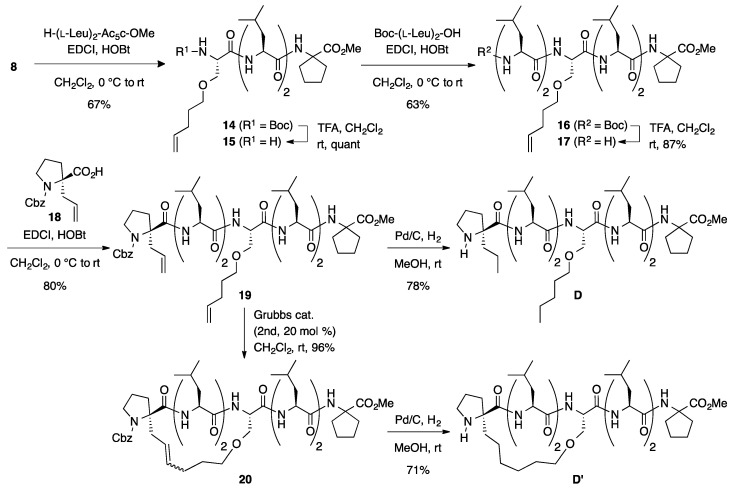
Synthesis of unstapled peptide **D** and stapled peptide **D’** (tethering at *i*, *i*+3 positions with an 18-membered ring).

The peptide stapling at the *i*,*i*+3 positions could be another choice to restrict the conformational freedom of the N-terminus. We designed spirocyclic-stapled peptide **D’** possessing tethered crosslinks at the *i*,*i*+3 positions by introducing (*R*)-α-allyl-proline **18** [52,53] (Scheme 3). The synthesis of **D’** began from *O*-(4-pentenyl)-l-serine **8**, which was sequentially coupled with H-l-Leu-l-Leu-Ac_5_c-OMe [44] on C-terminus and with Boc-l-Leu-l-Leu-OH followed by **18** on N-terminus to produce heptapeptide **19**. The ring-closing metathesis of **19** proceeded at high yield, but no *E*/*Z*-selectivity was observed (*E*/*Z* = 1.1:1.0). The poor selectivity may be caused by sterically congested (*R*)-α-allyl-proline. *N*-terminal-free heptapeptides **D** and **D’** were synthesized by the hydrogenolysis of **19** and **20**, respectively.

Next, we examined the Michael addition reaction of 1-methylindole (**22**) and α,β-unsaturated aldehyde **21** using 20 mol % of unstapled peptides **A**–**D** and stapled peptides **A’**–**D’** to compare their catalytic activities (Table 1). The reaction with stapled octapeptide **B’** showed a faster reaction rate than the reaction with stapled dipeptide **A’** (entries 2 and 4, 46% conversion after 6 d vs. 83% conversion after 1 d). This result suggests that the helical motif -[l-Leu-l-Leu-Ac_5_c]_2_- of stapled peptide **B’** is important to catalytic activity. Furthermore, stapled peptide **B’** is more active than unstapled peptide **B** (entries 3 and 4, 83% conversion with a 76% isolated yield vs. 12% conversion). Similar trends were observed for other stapled peptides **A’**, **C’**, and **D’**. Therefore, the introduction of side-chain stapling plays a key role in enhancing catalytic activity. Moderate ee values could be improved by peptide sequence screening. The absolute configuration of **23** was determined by comparisons of the chiral HPLC chart and the specific rotation with those in the literature [47,48].

The reaction with reduced catalyst loading (10 or 5 mol %) of stapled peptide **B’** displayed almost the same results as the reaction with 20 mol % (Table 2, entries 4 and 6), while that of unstapled peptide **B** resulted in further decreases in both the conversion yield and ee value (entries 3 and 5). Lowering the reaction temperature to 0 °C contributed to the deactivation of unstapled peptide **B** (entry 7), but the reaction with stapled peptide **B’** retained a high conversion, with increased ee values (entry 8). These results also support that side-chain hydrocarbon stapling enhances the catalytic activities in the reaction.

**Figure 2:**
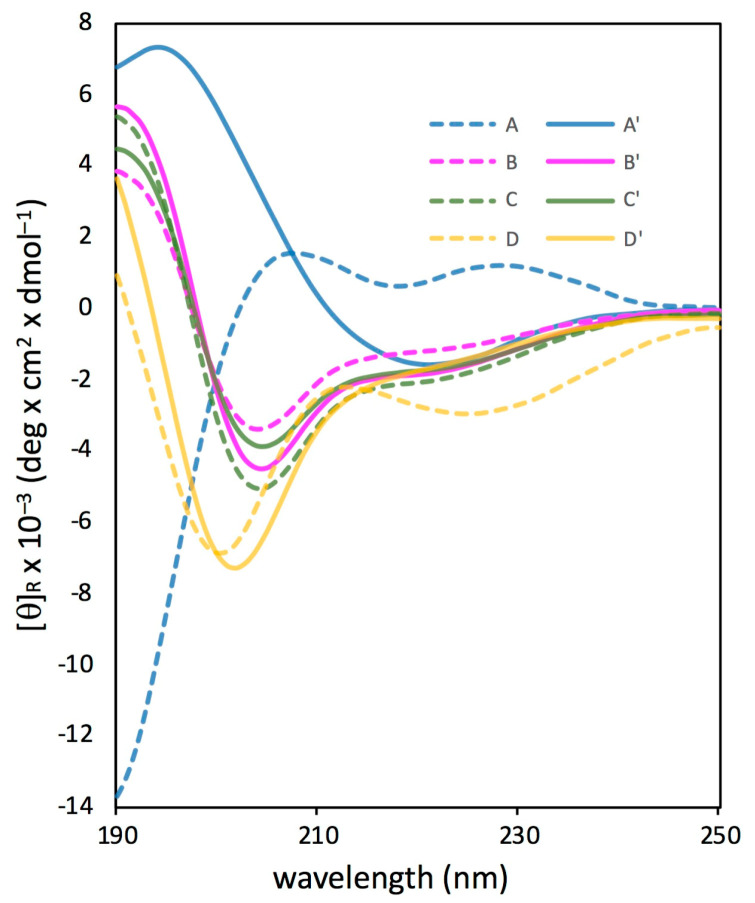
Circular dichroism (CD) spectra of peptide catalysts **A**–**D** and **A’**–**D’**. (0.5 mM in 2,2,2-trifluoroethanol).

Circular dichroism (CD) spectra were measured for all peptide catalysts to obtain their secondary structure information (Figure 2). The CD spectra of octapeptides **B**, **B’**, **C**, and **C’** and heptapeptides **D** and **D’** showed right-handed helical structures, while those of dipeptides **A** and **A’** showed β-turn structures. The helicity of stapled peptide **B’**, which gave the best conversion in Table 1, was higher than that of unstapled peptide **B**. These results suggest that reinforcement of helicity via side-chain stapling presumably increased the catalytic activity of the stapled peptide **B’**. Interestingly, the Michael reactions catalyzed by unstapled peptide **B** and stapled peptide **B’** produced opposite ee values, whereas both peptides showed right-handed helical structures. Therefore, the introduction of side-chain stapling to peptide catalysts can possibly reverse the enantioselectivities after the fine-tuning of the peptide sequence.

**Figure 3:**
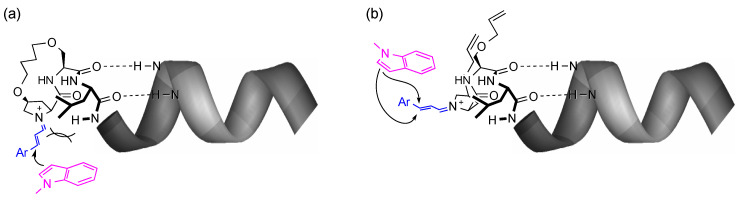
A plausible reaction mechanism catalyzed by (**a**) stapled peptide **B’** and (**b**) unstapled peptide **B**.

Based on the right-handed helical structure, the plausible reaction mechanism catalyzed by stapled peptide **B’** and unstapled peptide **B** is shown in Figure 3. For the reaction catalyzed by stapled peptide **B’**, the reactive iminium ion was formed inside the helical pipe with a rigid conformation caused by the hydrocarbon stapling, which accelerated the Friedel–Crafts type attack of 1-methylindole from the *si* face. On the other hand, the iminium species formed by unstapled peptide **B** exists outside the helical pipe with a flexible conformation, which decreased the reaction rate and enabled 1-methylindole to be accessed from both faces. Although the pioneering organocatalyst in this transformation, MacMillan’s imidazolidinone catalyst showed high enantioselectivities [54]; its derivatization as such a covalent immobilization to polymer supports as a recyclable catalyst is difficult and resulted in decreasing yield and enantioselectivities [55]. On the other hand, peptide catalysts are easier to modify and can be reused by immobilization in the resin at C-terminus, which does not affect the reactive site of N-terminus [21,22,23].

In summary, we have developed synthetic routes to the *N*-terminal l-prolyl oligopeptides **A’**–**D’** possessing side-chain hydrocarbon stapling. The ring-closing metathesis reactions of peptides **A** and **C** selectively produced *Z*- and *E*-configured stapled peptides, respectively, while no *E*/*Z*-selectivity was observed for the ring-closing metathesis of **D**. The stapled peptide **B’** catalyzed the Michael addition of 1-methylindole to α,β-unsaturated aldehyde, which was seven times faster than that of unstapled peptide **B**. Since the reactions with **B’** at lower catalyst loadings or lower temperatures retained conversion yields comparable to those of **B**, the introduction of side-chain hydrocarbon stapling is effective in enhancing the catalytic activity of peptides. These results provide useful information related to the recent progress of the *E*/*Z*-selective ring-closing metathesis of peptides [56,57], l-prolyl catalysts [58,59,60], and peptide foldamer [61,62,63]. Further studies including enantioselectivity improvement, an expansion of the reaction scope using catalyst **B’**, as well as applications to cell-penetrating peptides [64,65,66] are ongoing in our laboratory.

## 3. Materials and Methods

### 3.1. General Procedure and Method

Melting points were taken on an AS ONE melting point apparatus ATM-01 (AS ONE Corporation, Osaka, Japan) and were uncorrected. Optical rotations were measured on a JASCO DIP-370 polarimeter (JASCO Corporation, Tokyo, Japan) using CHCl_3_ as a solvent. ^1^H NMR and ^13^C NMR spectra were recorded on the JEOL JNM-AL-400 (400 MHz), a Varian NMR System 500PS SN (500 MHz and 125 MHz) spectrometer (Agilent Inc., Santa Clara, CA, USA). Chemical shifts (δ) are reported in parts per million (ppm). For the ^1^H NMR spectra (CDCl_3_), tetramethylsilane was used as the internal reference (0.00 ppm), while the central solvent peak was used as the reference (77.0 ppm in CDCl_3_) for the ^13^C NMR spectra. The IR spectra were recorded on a Shimadzu IRAffinity-1 FT-IR spectrophotometer (Shimadzu Corporation, Kyoto, Japan). High-resolution mass spectra (HRMS) were obtained on a JEOL JMS-T100TD using electrospray ionization (ESI) (JEOL Ltd., Tokyo, Japan) or direct analysis in the real-time (DART) ionization in time-of-flight (TOF) mode. Circular dichroism (CD) spectra were measured with a JASCO J-725N spectropolarimeter (JASCO Corporation, Tokyo, Japan) using a 1.0 mm path length cell. Analytical and semi-preparative thin layer chromatography (TLC) was performed with Merck Millipore pre-coated TLC plates (MilliporeSigma, Burlington, NJ, USA), silica gel 60 F_254_, and layer thicknesses of 0.25 and 0.50 mm, respectively. Compounds were observed in UV light at 254 nm and then visualized by staining with iodine, *p*-anisaldehyde, or phosphomolybdic acid stain. Flash and gravity column chromatography separations were performed on Kanto Chemical silica gel 60N, spherical neutral, with particle sizes of 63–210 μm and 40–50 μm, respectively. Analytical high-performance liquid chromatography (HPLC) was carried out with JASCO PU-2089 on a UV spectrophotometric detector (254 nm, JASCO UV-2075, JASCO Corporation, Tokyo, Japan), to which a 4.6 × 250 mm size chiral column (Daicel Chiralpak AD-H, Daicel Corporation, Osaka, Japan) was attached. All moisture-sensitive reactions were conducted under an inert atmosphere. Reagents and solvents were of commercial grade and were used as supplied, unless otherwise noted. Compounds **1** [49,50], **2** [24,25], **8** [51], **18** [52,53], H-(l-Leu-l-Leu-Ac_5_c)_2_-OMe [44], and H-l-Leu-l-Leu-Ac_5_c-OMe [44] were prepared according to the reported procedures. Copies of NMR Spectra are given in the Supplementary Materials.

### 3.2. Synthesis of Unstapled Peptides A and B and Stapled Peptides A’ and B’

*Boc-l-Hyp^OAll^-l-Ser^OAll^-OMe* (**3**): To a solution of Boc-l-Hyp^OAll^-OH (**1** [49,50]; 4.22 g, 15.5 mmol) in CH_2_Cl_2_ (52 mL) were added 3-[bis(dimethylamino)methyliumyl]-3*H*-benzotriazol-1-oxide hexafluorophosphate (HBTU; 6.48 g, 17.1 mmol) and 1-hydroxybenzotriazole hydrate (HOBt·H_2_O; 2.62 g, 17.1 mmol) at 0 °C, and the solution was stirred for 30 min. Then, a solution of HCl·H-l-Ser^OAll^-OMe (**2** [24,25].; 2.47 g, 15.5 mmol) in CH_2_Cl_2_ (52 mL) and *N*,*N*-diisopropylethylamine (DIPEA; 5.41 mL, 31.1 mmol) was added to the reaction mixture at the same temperature, and the resultant mixture was gradually warmed to room temperature. After stirring overnight, the CH_2_Cl_2_ was removed and the residue was diluted with EtOAc. The solution was washed successively with 1 M of HCl, water, sat. aq NaHCO_3_, and brine. The organic layer was dried over anhydrous MgSO_4_ and concentrated in vacuo to give a crude product, which was purified by flash column chromatography on silica gel (40% EtOAc in *n*-hexane) to give **3** (3.89 g, 61%) as a yellow oil. *R*_f_ = 0.69 (EtOAc). ${\left[\alpha \right]}_{D}^{30}$ −3.2 (*c* 1.02, CHCl_3_). ^1^H NMR (500 MHz, CDCl_3_, VT = 50 °C) δ: 5.98–5.77 (m, 2H), 5.28–5.11 (m, 4H), 4.77–4.66 (m, 1H), 4.33 (d, *J* = 8.8 Hz, 1H), 4.07–4.04 (m, 1H), 4.00–3.86 (m, 4H), 3.82 (dd, *J* = 9.7, 3.5, Hz, 1H), 3.78–3.70 (m, 1H), 3.74 (s, 3H), 3.61–3.49 (m, 3H), 2.56–2.44 (m, 1H), 2.23–2.09 (m, 1H), 1.48 (s, 9H). ^13^C NMR (125 MHz, CDCl_3_, VT = 50 °C) δ: 172.0, 170.5, 156.0, 134.6, 134.3, 117.0, 116.9, 81.0, 76.4, 72.2, 69.9, 69.6, 58.6, 53.0, 52.8, 52.2, 37.0, 28.3 (3C). IR (film): 3304, 2978, 2933, 1751, 1701 cm^−1^. HRMS (DART) *m*/*z*: [M + H]^+^ calcd for C_20_H_33_N_2_O_7_, 413.2288; found, 413.2282.

*H-l-Hyp^OAll^-l-Ser^OAll^-OMe* (**A**): To a solution of Boc-protected dipeptide **3** (100 mg, 0.242 mmol) in CH_2_Cl_2_ (2.4 mL) was added trifluoroacetic acid (0.24 mL) dropwise at room temperature, and the reaction mixture was stirred for 2 days at the same temperature. The reaction mixture was neutralized by adding sat. aq NaHCO_3_ and the aqueous phase was extracted with CHCl_3_ three times. The combined organic extracts were dried over anhydrous MgSO_4_ and concentrated under a vacuum to give amine **A** (75.2 mg, quant) as an amorphous solid. *R*_f_ = 0.29 (EtOAc). Mp 81–83 °C. ${\left[\alpha \right]}_{D}^{28}$ −39.4 (*c* 1.00, CHCl_3_). ^1^H NMR (400 MHz, CDCl_3_) δ: 8.27 (d, *J* = 8.6 Hz, 1H), 5.93–5.78 (m, 2H), 5.29–5.21 (m, 2H), 5.21–5.11 (m, 2H), 4.72 (dt, *J* = 8.3, 3.5 Hz, 1H), 4.08–4.00 (m, 1H), 4.00–3.96 (m, 2H), 3.94 (dt, *J* = 5.4, 1.5 Hz, 1H), 3.92–3.85 (m, 2H), 3.82 (dd, *J* = 8.3, 4.9 Hz, 1H), 3.76 (s, 3H), 3.58 (dd, *J* = 9.5, 3.7 Hz, 1H), 3.17 (dd, *J* = 11.2, 5.4 Hz, 1H), 3.05 (dd, *J* = 11.2, 2.9 Hz, 1H), 2.28–2.16 (m, 2H). ^13^C NMR (125 MHz, CDCl_3_) δ: 168.4, 163.9, 134.1, 133.6, 118.0, 117.4, 74.6, 72.3, 70.1, 68.5, 57.2, 54.1, 52.5, 50.7, 33.6. IR (KBr): 3215, 2874, 1692, 1450 cm^−1^. HRMS (ESI) *m*/*z*: [M + Na]^+^ calcd for C_15_H_24_N_2_O_5_Na, 335.1583; found, 335.1573.

*Boc-l-Hyp^OAll^-l-Ser^OAll^-[(l-Leu)_2_-Ac_5_c]_2_-OMe* (**4**): To a solution of dipeptide **3** (50.0 mg, 0.121 mmol) in MeOH (1.2 mL) was added 1 M of aqueous NaOH (0.121 mL, 0.121 mmol) at room temperature, and the mixture was stirred overnight at the same temperature. The solution was acidified with 1 M of aqueous HCl and the MeOH was removed in vacuo. The resulting aqueous solution was extracted with EtOAc three times. The combined organic extracts were washed with brine, dried over Na_2_SO_4_, and concentrated in vacuo to give a carboxylic acid (44.7 mg, 93%). To a solution of the acid (206 mg, 0.500 mmol) in CH_2_Cl_2_ (2.5 mL) was added EDCI·HCl (96.0 mg, 0.500 mmol) and HOBt·H_2_O (92.0 mg, 0.600 mmol) at 0 °C, and the mixture was stirred at the same temperature for 30 min. Then, a solution of H-[(l-Leu)_2_-Ac_5_c]_2_-OMe (354 mg, 0.500 mmol) in CH_2_Cl_2_ (2.5 mL) was added dropwise to the reaction mixture at 0 °C. The reaction mixture was gradually warmed to room temperature and stirred overnight. After the removal of CH_2_Cl_2_, the residue was diluted with EtOAc. The solution was washed successively with 1 M of HCl, water, sat. aq NaHCO_3_, and brine. The organic layer was dried over anhydrous MgSO_4_ and concentrated in vacuo to give crude product, which was purified by flash column chromatography on silica gel (70% EtOAc in *n*-hexane) to give **4** (269 mg, 49%) as a white solid. *R*_f_ = 0.20 (60% EtOAc in *n*-hexane). Mp 76–79 °C. ${\left[\alpha \right]}_{D}^{27}$ +2.2 (*c* 1.02, CHCl_3_). ^1^H NMR (500 MHz, CDCl_3_) δ: 7.49 (d, *J* = 5.6 Hz, 1H), 7.45 (d, *J* = 7.8 Hz, 1H), 7.39 (d, *J* = 4.9 Hz, 1H), 7.28–7.23 (m, 4H), 5.96–5.74 (m, 2H), 5.37–5.16 (m, 4H), 4.34 (t, *J* = 8.6 Hz, 1H), 4.25–4.12 (m, 5H), 4.05–3.90 (m, 5H), 3.84–3.73 (m, 2H), 3.72–3.65 (m, 1H), 3.67 (s, 3H), 3.48 (dd, *J* = 12.0, 3.4 Hz, 1H), 2.70–2.60 (m, 1H), 2.39–2.22 (m, 3H), 2.22–2.11 (m, 3H), 2.11–2.02 (m, 1H), 1.96–1.57 (m, 22H), 1.50 (s, 9H), 1.02–0.83 (m, 24H). ^13^C NMR (125 MHz, CDCl_3_) δ: 175.6, 175.2, 174.6, 174.2, 174.1, 173.2, 173.1, 171.8, 155.7, 133.9, 133.5, 117.8, 117.5, 81.7, 72.2, 69.6, 67.6, 66.7, 65.7, 60.1, 56.2, 54.8, 54.1, 54.0, 53.3, 52.23, 52.22, 52.1, 39.6, 39.4, 39.1, 38.3, 37.3, 36.74, 36.70, 35.5, 34.4, 28.32, 28.26 (3C), 25.2, 25.0, 24.73, 24.65, 24.54, 24.53, 24.46, 23.5, 23.4, 22.99, 22.97, 21.1, 21.0, 20.90, 20.87. IR (CDCl_3_): 3325, 2961, 1732, 1661, 1530 cm^−1^. HRMS (ESI) *m*/*z*: [M + Na]^+^ calcd for C_56_H_94_N_8_O_13_Na, 1109.6838; found, 1109.6808.

*H-l-Hyp^OAll^-l-Ser^OAll^-[(l-Leu)_2_-Ac_5_c]_2_-OMe* (**B**): To a solution of Boc-protected peptide **4** (135 mg, 0.124 mmol) in CH_2_Cl_2_ (1 mL) was added trifluoroacetic acid (0.12 mL) dropwise at room temperature, and the reaction mixture was stirred overnight at the same temperature. The reaction mixture was neutralized by adding sat. aq NaHCO_3_ and the aqueous phase was extracted with CHCl_3_ four times. The combined organic extracts were dried over anhydrous MgSO_4_ and concentrated under vacuum to give amine product **B** (124 mg, quant). *R*_f_ = 0.10 (80% EtOAc in *n*-hexane). Mp 107–108 °C. ${\left[\alpha \right]}_{D}^{30}$ −1.9 (*c* 0.99, CHCl_3_). ^1^H NMR (500 MHz, CDCl_3_) δ: 9.04 (br s, 1H), 8.06 (br s, 1H), 7.64 (d, *J* = 6.8 Hz, 1H), 7.57 (br s, 1H), 7.52–7.42 (m, 2H), 7.35 (d, *J* = 4.9 Hz, 1H), 5.92–5.75 (m, 2H), 5.32–5.14 (m, 4H), 4.60 (br s, 1H), 4.35 (d, *J* = 4.2 Hz, 1H), 4.29 (br s, 1H), 4.21–4.04 (m, 3H), 4.04–3.95 (m, 4H), 3.95–3.88 (m, 1H), 3.86–3.74 (m, 2H), 3.68 (s, 3H), 3.60 (d, *J* = 11.7 Hz, 1H), 3.47 (d, *J* = 8.8 Hz, 1H), 2.65–2.44 (m, 3H), 2.29–2.16 (m, 2H), 2.12 (br s, 2H), 2.03 (dd, *J* = 11.7, 6.1 Hz, 1H), 1.92–1.53 (m, 22H), 1.03–0.78 (m, 24H). ^13^C NMR (125 MHz, CDCl_3_) δ: 175.7, 175.5, 175.4, 174.7, 174.4, 174.3, 173.9, 171.8, 133.8, 133.5, 118.0, 117.9, 76.1, 72.3, 70.0, 68.1, 66.7, 66.0, 59.3, 56.4, 55.0, 54.9, 54.4, 53.3, 52.4, 51.3, 40.1, 39.7, 39.4, 39.2, 37.9, 36.89, 36.85, 35.2, 35.0, 29.7, 25.0, 24.78, 24.76, 24.58, 24.56, 24.4, 24.3, 24.23, 24.17, 23.2, 23.1, 22.5, 21.8, 21.6, 21.2. IR (KBr): 3329, 2959, 1736, 1655, 1535 cm^−1^. HRMS (ESI) *m*/*z*: [M + Na]^+^ calcd for C_51_H_86_N_8_O_11_Na, 1009.6314; found, 1009.6288.

*Stapled Boc-l-Hyp-l-Ser-OMe* (**6**): Under an argon atmosphere, to a solution of **3** (90.0 mg, 0.218 mmol) in CH_2_Cl_2_ (11 mL) was added second-generation Grubbs catalyst (37.0 mg, 0.0436 mmol) at room temperature, and the mixture was stirred for 2 h at the same temperature. The reaction mixture was filtered through a short pad of silica gel (60% EtOAc in *n*-hexane) and concentrated. The crude material was purified by flash chromatography on silica gel (60% EtOAc in *n*-hexane) to provide a stapled peptide **5** (46.2 mg, 55%) as a mixture of *E*- and *Z*-isomers (*E*/*Z* = 1.0:5.6). *R*_f_ = 0.30 (EtOAc). Next, to a solution of stapled peptides **5** (46.2 mg, 0.120 mmol) in MeOH (12 mL) was added 10% Pd-C (23 mg, 50 wt %) under a nitrogen atmosphere. After being vigorously stirred under a hydrogen atmosphere for 19 h at room temperature, the reaction mixture was passed through a short plug of Celite. The filtrate was concentrated under vacuum to give a crude product, which was purified by flash column chromatography on silica gel (70% EtOAc in *n*-hexane) to give **6** (35.5 mg, 77%) as an amber oil. *R*_f_ = 0.29 (EtOAc). ${\left[\alpha \right]}_{D}^{29}$ −3.5 (*c* 1.07, CHCl_3_). ^1^H NMR (500 MHz, CDCl_3_) δ: 7.27 (br s, 1H), 4.76–4.56 (m, 1H), 4.39–4.21 (m, 1H), 3.97 (br s, 2H), 3.87–3.78 (m, 1H), 3.76 (s, 3H), 3.59 (br s, 3H), 3.54–3.39 (m, 2H), 3.38–3.29 (m, 1H), 2.40–2.17 (m, 2H), 1.92–1.77 (m, 2H), 1.77–1.54 (m, 2H), 1.54–1.38 (m, 9H). ^13^C NMR (125 MHz, CDCl_3_) δ: 172.5, 170.3, 154.9, 80.8, 70.8, 69.7, 69.5, 69.1, 60.6, 53.0, 52.4, 52.0, 37.2, 28.1 (3C), 26.9, 25.5. IR (film): 3422, 2934, 1751, 1697 cm^−1^. HRMS (DART) *m*/*z*: [M + H]^+^ calcd for C_18_H_31_N_2_O_7_, 387.2131; found, 387.2130.

*Stapled H-l-Hyp-l-Ser-OMe* (**A’**): To a solution of Boc-protected dipeptide **6** (45.0 mg, 0.116 mmol) in CH_2_Cl_2_ (1 mL) was added trifluoroacetic acid (0.2 mL) dropwise at room temperature, and the reaction mixture was stirred for 24 h at the same temperature. The reaction mixture was neutralized by adding sat. aq NaHCO_3_, and the aqueous phase was extracted with CHCl_3_ three times. The combined organic extracts were dried over anhydrous MgSO_4_ and concentrated under vacuum to give crude product **A’** (20.5 mg, 62%) as an amber oil, which was used for the next step without further purification. *R*_f_ = 0.30 (EtOAc). ${\left[\alpha \right]}_{D}^{28}$ +11.6 (*c* 1.00, CHCl_3_). ^1^H NMR (500 MHz, CDCl_3_) δ: 8.52 (d, *J* = 7.6 Hz, 1H), 4.69–4.63 (m, 1H), 3.95 (t, *J* = 3.8 Hz, 1H), 3.91–3.82 (m, 2H), 3.78 (s, 3H), 3.79–3.74 (m, 1H), 3.61 (dt, *J* = 9.8, 5.9 Hz, 1H), 3.53–3.43 (m, 2H), 3.43–3.31 (m, 2H), 2.99 (dd, *J* = 10.5, 3.2 Hz, 1H), 2.27 (d, *J* = 14.2 Hz, 1H), 2.15 (ddd, *J* = 14.2, 11.0, 4.2 Hz, 1H), 1.78–1.46 (m, 4H). ^13^C NMR (125 MHz, CDCl_3_) δ: 175.1, 171.0, 78.6, 71.1, 69.0, 68.3, 58.9, 53.4, 52.5, 51.3, 37.0, 26.5, 26.1. IR (KBr): 3345, 2920, 2868, 1748, 1658, 1526, 1441 cm^−1^. HRMS (ESI) *m*/*z*: [M + Na]^+^ calcd for C_13_H_22_N_2_O_5_Na, 309.1426; found, 309.1428.

*Stapled Boc-l-Hyp-l-Ser-[(l-Leu)_2_-Ac_5_c]_2_-OMe* (**7**): To a solution of stapled dipeptide **6** (104 mg, 0.269 mmol) in MeOH (3 mL) was added 1 M of aqueous NaOH (0.270 mL, 0.270 mmol) at room temperature, and the mixture was stirred overnight at the same temperature. The solution was acidified with 1 M of aqueous HCl and the MeOH was removed in vacuo. The resulting aqueous solution was extracted with EtOAc three times. The combined organic extracts were washed with brine, dried over Na_2_SO_4_, and concentrated in vacuo to give a crude product (95.0 mg, 95%), which was used for the next step without further purification. *R*_f_ = 0.27 (EtOAc). To a solution of the crude acid (85.6 mg, 0.230 mmol) in CH_2_Cl_2_ (2.3 mL) was added *N*-(3-dimethylaminopropyl)-*N’*-ethylcarbodiimide hydrochloride (EDCI·HCl; 44.0 mg, 0.230 mmol) and HOBt·H_2_O (42.0 mg, 0.276 mmol) at 0 °C, and the mixture was stirred at the same temperature for 30 min. Then, a solution of H-[(l-Leu)_2_-Ac_5_c]_2_-OMe [44] (163 mg, 0.230 mmol) in CH_2_Cl_2_ (1 mL) was added dropwise to the reaction mixture at 0 °C. The reaction mixture was gradually warmed to room temperature and stirred for 2 days. After the removal of CH_2_Cl_2_, the residue was diluted with EtOAc. The solution was washed successively with 1 M of HCl, water, sat. aq NaHCO_3_, and brine. The organic layer was dried over anhydrous MgSO_4_ and concentrated in vacuo to give a crude product, which was purified by flash column chromatography on silica gel (80% EtOAc in *n*-hexane) to give **7** (178 mg, 73%) as a yellow oil. *R*_f_ = 0.46 (EtOAc). ${\left[\alpha \right]}_{D}^{28}$ −4.1 (*c* 1.07, CHCl_3_). ^1^H NMR (500 MHz, CDCl_3_) δ: 7.75 (br s, 1H), 7.54–7.41 (m, 2H), 7.31 (s, 1H), 7.28–7.25 (m, 2H), 7.22 (d, *J* = 6.1 Hz, 1H), 4.37–4.30 (m, 1H), 4.28 (dd, *J* = 10.5, 4.2 Hz, 1H), 4.24–4.16 (m, 2H), 4.14 (d, *J* = 10.3 Hz, 1H), 4.08–4.03 (m, 1H), 3.98 (dd, *J* = 11.0, 4.9 Hz, 1H), 3.96–3.90 (m, 1H), 3.84 (d, *J* = 11.7 Hz, 1H), 3.75–3.69 (m, 1H), 3.67 (s, 3H), 3.65–3.50 (m, 3H), 3.46–3.36 (m, 2H), 2.70–2.60 (m, 1H), 2.39 (ddd, *J* = 14.6, 10.9, 4.0 Hz, 1H), 2.31–2.03 (m, 8H), 1.94–1.59 (m, 24H), 1.52 (s, 9H), 1.01–0.84 (m, 24H). ^13^C NMR (125 MHz, CDCl_3_) δ: 175.4, 175.2 (2C), 174.4, 174.2, 173.2, 173.1, 171.0, 155.4, 81.4, 78.9, 70.8, 68.5, 68.4, 66.7, 65.7, 60.4, 54.9, 54.7, 54.0, 53.9, 53.4, 52.2, 52.0, 40.0, 39.6, 39.4, 39.2, 38.2, 37.2, 36.7, 35.4, 35.3, 28.3 (3C), 27.4, 26.3, 25.0, 24.82, 24.81, 24.6, 24.50 (2C), 24.48, 24.4, 23.5, 23.4, 22.9, 22.6, 21.5, 21.2, 21.0, 20.9. IR (KBr): 3329, 2957, 1736, 1647, 1522 cm^−1^. HRMS (ESI) *m*/*z*: [M + Na]^+^ calcd for C_54_H_92_N_8_O_13_Na, 1083.6682; found, 1083.6685.

*Stapled H-l-Hyp-l-Ser-[(l-Leu)_2_-Ac_5_c]_2_-OMe* (**B’**): To a solution of Boc-protected peptide **7** (120 mg, 0.113 mmol) in CH_2_Cl_2_ (2 mL) was added trifluoroacetic acid (0.113 mL) dropwise at room temperature, and the reaction mixture was stirred overnight at the same temperature. The reaction mixture was neutralized by adding sat. aq NaHCO_3_, and the aqueous phase was extracted with CHCl_3_ three times. The combined organic extracts were dried over anhydrous MgSO_4_ and concentrated under a vacuum to give crude product **B’** (96.9 mg, 89%), which was used for the next step without further purification. *R*_f_ = 0.25 (EtOAc). Mp 117–118 °C. ${\left[\alpha \right]}_{D}^{26}$ −5.2 (*c* 0.95, CHCl_3_). ^1^H NMR (500 MHz, CDCl_3_) δ: 8.83 (d, *J* = 5.4 Hz, 1H), 7.58 (d, *J* = 4.6 Hz, 1H), 7.45 (d, *J* = 7.8 Hz, 1H), 7.34 (s, 1H), 7.32–7.24 (m, 3H), 4.37–4.27 (m, 2H), 4.23–4.16 (m, 1H), 4.11 (br s, 2H), 4.02 (dd, *J* = 10.9, 5.0 Hz, 1H), 3.94 (dt, *J* = 9.7, 4.8 Hz, 1H), 3.89 (dd, *J* = 8.8, 3.4 Hz, 1H), 3.78 (dd, *J* = 10.9, 3.1 Hz, 1H), 3.67 (s, 3H), 3.66–3.61 (m, 1H), 3.60–3.50 (m, 3H), 3.34 (d, *J* = 10.5 Hz, 1H), 3.06 (dd, *J* = 10.5, 2.4 Hz, 1H), 2.69–2.60 (m, 1H), 2.36 (br s, 3H), 2.30–2.21 (m, 4H), 2.20–2.03 (m, 3H), 1.97–1.55 (m, 24H), 1.03–0.80 (m, 24H). ^13^C NMR (125 MHz, CDCl_3_) δ: 178.1, 175.6, 175.1, 174.2, 173.9, 173.2, 173.1, 172.4, 80.0, 70.3, 69.8, 68.7, 66.8, 65.7, 59.8, 55.2, 54.8, 54.1, 54.0, 52.3, 52.1, 51.5, 39.6, 39.3, 38.2, 37.2, 36.7, 36.3, 35.4, 29.6, 28.4, 27.2, 26.5, 25.2, 25.12, 25.07, 24.7, 24.50, 24.49, 24.4 (2C), 23.5, 23.4, 23.1, 22.8, 21.3, 21.1, 21.0, 20.8. IR (CDCl_3_): 3325, 2958, 1655, 1526 cm^−1^. HRMS (ESI) *m*/*z*: [M + Na]^+^ calcd for C_49_H_84_N_8_O_11_Na, 983.6157; found, 983.6142.

### 3.3. Synthesis of Unstapled Peptide C and Stapled Peptide C’

*Boc-l-Ser^OPte^-[(l-Leu)_2_-Ac_5_c]_2_-OMe* (**9**): To a solution of Boc-l-Ser^OPte^-OH **8** [51] (193 mg, 0.707 mmol) in CH_2_Cl_2_ (2.5 mL) were added EDCI·HCl (136 mg, 0.707 mmol) and HOBt·H_2_O (130 mg, 0.848 mmol) at 0 °C, and the solution was stirred for 30 min. Then, a solution of H-[(l-Leu)_2_-Ac_5_c]_2_-OMe [44] (500 mg, 0.707 mmol) in CH_2_Cl_2_ (2.5 mL) was added to the reaction mixture at the same temperature, and the resultant mixture was gradually warmed to room temperature. After stirring overnight, CH_2_Cl_2_ was removed, and the residue was diluted with EtOAc. The solution was washed successively with 1 M of HCl, water, sat. aq NaHCO_3_, and brine. The organic layer was dried over anhydrous MgSO_4_ and concentrated in vacuo to give a crude product, which was purified by flash column chromatography on silica gel (60% EtOAc in *n*-hexane) to give **9** (478 mg, 70%) as a white solid. *R*_f_ = 0.66 (EtOAc). Mp 109–115 °C. ${\left[\alpha \right]}_{D}^{26}$ −4.3 (*c* 1.00, CHCl_3_). ^1^H NMR (500 MHz, CDCl_3_) δ: 7.42 (d, *J* = 8.1 Hz, 1H), 7.33 (s, 1H), 7.29 (d, *J* = 4.4 Hz, 1H), 7.25–7.19 (m, 2H), 6.60 (d, *J* = 3.7 Hz, 1H), 5.80 (ddt, *J* = 17.0, 10.3, 6.6 Hz, 1H), 5.50 (d, *J* = 2.4 Hz, 1H), 5.08–4.96 (m, 2H), 4.39–4.32 (m, 1H), 4.20 (dd, *J* = 11.2, 6.1 Hz, 1H), 4.14–4.07 (m, 1H), 4.04 (q, *J* = 3.7 Hz, 1H), 3.98 (dt, *J* = 9.7, 5.0, Hz, 1H), 3.77–3.70 (m, 2H), 3.67 (s, 3H), 3.55–3.44 (m, 2H), 2.70–2.60 (m, 1H), 2.32–2.23 (m, 1H), 2.23–2.02 (m, 6H), 1.96–1.54 (m, 24H), 1.50 (s, 9H), 1.05–0.82 (m, 24H). ^13^C NMR (125 MHz, CDCl_3_) δ: 175.5, 175.1, 173.6, 173.4, 173.0, 172.9, 172.0, 156.9, 137.7, 115.1, 81.7, 71.0, 68.8, 66.8, 65.7, 56.9, 54.7, 54.1, 54.0, 52.2, 52.1, 40.1, 39.8, 39.6, 39.4, 38.1, 37.3, 36.7, 35.5, 30.2, 28.5, 28.1 (3C), 25.3, 25.1, 24.8, 24.7, 24.53, 24.51, 24.43, 24.40, 23.5, 23.4, 23.0, 22.9, 21.4, 21.3, 21.1, 20.9. IR (KBr): 3329, 2957, 1701, 1632, 1524 cm^−1^. HRMS (ESI) *m*/*z*: [M + Na]^+^ calcd for C_50_H_87_N_7_O_11_Na, 984.6361; found, 984.6386.

*Boc-l-Hyp^OAll^-l-Ser^OPte^-[(l-Leu)_2_-Ac_5_c]_2_-OMe* (**11**): To a solution of Boc-protected peptide **9** (480 mg, 0.499 mmol) in CH_2_Cl_2_ (5 mL) was added trifluoroacetic acid (0.749 mL) dropwise at room temperature, and the reaction mixture was stirred overnight at the same temperature. The reaction mixture was neutralized by adding sat. aq NaHCO_3_, and the aqueous phase was extracted with CHCl_3_ three times. The combined organic extracts were dried over anhydrous MgSO_4_ and concentrated under a vacuum to give crude product **10** (391 mg, 91%), which was used for the next step without further purification. *R*_f_ = 0.20 (60% EtOAc in *n*-hexane). To a solution of Boc-l-Hyp^OAll^-OH (123 mg, 0.454 mmol) in CH_2_Cl_2_ (2.5 mL) were added EDCI·HCl (87.0 mg, 0.454 mmol) and HOBt·H_2_O (84.0 mg, 0.545 mmol) at 0 °C, and the solution was stirred for 30 min. Then, a solution of amine **10** (391 mg, 0.454 mmol) in CH_2_Cl_2_ (2.5 mL) was added to the reaction mixture at the same temperature, and the resultant mixture was gradually warmed to room temperature. After stirring overnight, the CH_2_Cl_2_ was removed and the residue was diluted with EtOAc. The solution was washed successively with 1 M of HCl, water, sat. aq NaHCO_3_, and brine. The organic layer was dried over anhydrous MgSO_4_ and concentrated in vacuo to give crude product, which was purified by flash column chromatography on silica gel (70% EtOAc in *n*-hexane) to give **11** (248 mg, 49%) as a white solid. *R*_f_ = 0.56 (EtOAc). Mp 75–85 °C. ${\left[\alpha \right]}_{D}^{20}$ −3.2 (*c* 1.03, CHCl_3_). ^1^H NMR (500 MHz, CDCl_3_) δ: 7.49–7.41 (m, 2H), 7.37 (d, *J* = 4.9 Hz, 1H), 7.26–7.20 (m, 4H), 5.93–5.72 (m, 2H), 5.33–5.19 (m, 2H), 5.06–4.95 (m, 2H), 4.39–4.30 (m, 1H), 4.25–4.09 (m, 5H), 4.05–3.90 (m, 3H), 3.83–3.72 (m, 2H), 3.67 (s, 3H), 3.67–3.63 (m, 1H), 3.52–3.38 (m, 3H), 2.70–2.61 (m, 1H), 2.36–2.32 (m, 1H), 2.31–2.11 (m, 4H), 2.10–2.02 (m, 3H), 1.96–1.66 (m, 21 H), 1.66–1.56 (m, 4H), 1.51 (s, 9H), 1.03–0.80 (m, 24H). ^13^C NMR (125 MHz, CDCl_3_) δ: 175.6, 175.2, 174.6, 174.2, 174.1, 173.1, 173.0, 171.9, 155.7, 137.5, 133.9, 117.5, 115.2, 81.6, 77.2, 70.8, 69.6, 68.3, 66.8, 65.7, 60.2, 56.2, 54.8, 54.1, 54.0, 53.4, 52.2, 52.1, 39.7, 39.6, 39.4, 39.2, 38.3, 37.3, 36.7, 35.5, 34.3, 30.0, 28.4, 28.3 (3C), 25.2, 25.1, 24.8, 24.7, 24.57, 24.55 (2C), 24.47, 23.5, 23.4, 23.00, 22.97, 21.12, 21.06, 21.0, 20.9. IR (CDCl_3_): 3325, 2959, 2872, 1732, 1661, 1530 cm^−1^. HRMS (ESI) *m*/*z*: [M + Na]^+^ calcd for C_58_H_98_N_8_O_13_Na, 1137.7151; found, 1137.7201.

*H-l-Hyp^OAll^-l-Ser^OPte^-[(l-Leu)_2_-Ac_5_c]_2_-OMe* (**C**): To a solution of Boc-protected peptide **11** (30.0 mg, 0.0269 mmol) in CH_2_Cl_2_ (1 mL) was added trifluoroacetic acid (0.03 mL) dropwise at room temperature, and the reaction mixture was stirred overnight at the same temperature. The reaction mixture was neutralized by adding sat. aq NaHCO_3_, and the aqueous phase was extracted with CHCl_3_ three times. The combined organic extracts were dried over anhydrous MgSO_4_ and concentrated under a vacuum to give crude product **C** (23.5 mg, 86%), which was used for the next step without further purification. *R*_f_ = 0.31 (EtOAc). Mp 79–81 °C. ${\left[\alpha \right]}_{D}^{28}$ −7.8 (*c* 1.00, CHCl_3_). ^1^H NMR (500 MHz, CDCl_3_) δ: 8.45 (br s, 1H), 7.65 (d, *J* = 5.1 Hz, 1H), 7.46 (d, *J* = 7.8 Hz, 1H), 7.32 (s, 1H), 7.26–7.21 (m, 2H), 6.59 (br s, 1H), 5.90–5.74 (m, 2H), 5.27–5.12 (m, 2H), 5.08–4.95 (m, 2H), 4.37–4.26 (m, 1H), 4.25–4.15 (m, 1H), 4.15–4.08 (m, 2H), 4.04 (br s, 1H), 3.98–3.83 (m, 4H), 3.76 (dd, *J* = 10.0, 4.0 Hz, 1H), 3.70 (dd, *J* = 10.0, 3.9 Hz, 1H), 3.67 (s, 3H), 3.55–3.43 (m, 2H), 3.20 (dd, *J* = 11.3, 4.2 Hz, 1H), 3.13 (d, *J* = 11.3 Hz, 1H), 2.64 (dt, *J* = 13.6, 8.4 Hz, 1H), 2.37 (d, *J* = 13.7 Hz, 1H), 2.30–2.02 (m, 8H), 1.98–1.50 (m, 25H), 1.03–0.81 (m, 24H). ^13^C NMR (125 MHz, CDCl_3_) δ: 178.3, 175.6, 175.2, 174.2, 174.1, 173.3, 173.2, 172.1, 137.7, 134.6, 116.8, 115.1, 78.7, 70.7, 69.6, 68.8, 66.8, 65.7, 59.2, 56.5, 54.8, 54.1 (2C), 52.4, 52.3, 52.1, 39.64, 39.62, 39.4, 39.1, 38.2, 37.2, 36.7, 35.7, 35.3, 30.1, 28.6, 25.2, 25.0 (2C), 24.7, 24.52, 24.49, 24.4, 24.3, 23.45, 23.36, 22.99, 22.96, 21.13, 21.08 (2C), 20.9. IR (KBr): 3343, 2957, 1639, 1547 cm^−1^. HRMS (ESI) *m*/*z*: [M + H]^+^ calcd for C_53_H_91_N_8_O_11_, 1015.6807; found, 1015.6843.

*Stapled Boc-l-Hyp-l-Ser-[(l-Leu)_2_-Ac_5_c]_2_-OMe* (**13**): Under an argon atmosphere, to a solution of **11** (70.0 mg, 0.0628 mmol) in CH_2_Cl_2_ (3 mL) was added second-generation Grubbs catalyst (10.7 mg, 0.0126 mmol) at room temperature, and the mixture was stirred for 2 h at the same temperature. The reaction mixture was filtered through short pad of silica gel (EtOAc) and concentrated. The crude material was purified by flash chromatography on silica gel (70% EtOAc in *n*-hexane) to provide a stapled peptide **12** (63.2 mg, 93%) as a mixture of *E*- and *Z*-isomers (*E*/*Z* = 5.5:1). *R*_f_ = 0.43 (EtOAc). Next, to a solution of stapled peptides **12** (52.9 mg, 0.0486 mmol) in MeOH (4 mL) was added 10% Pd-C (26 mg, 50 wt %) under a nitrogen atmosphere. After being vigorously stirred under a hydrogen atmosphere for 23 h at room temperature, the reaction mixture was passed through a short plug of Celite. The filtrate was concentrated under vacuum to give a crude product, which was purified by flash column chromatography on silica gel (4% MeOH in CHCl_3_) to give **13** (46.9 mg, 89%) as a colorless oil. *R*_f_ = 0.13 (3% MeOH in CHCl_3_). ${\left[\alpha \right]}_{D}^{25}$ −7.8 (*c* 1.00, CHCl_3_). ^1^H NMR (500 MHz, CDCl_3_) δ: 7.58 (d, *J* = 5.9 Hz, 1H), 7.50 (d, *J* = 4.9 Hz, 1H), 7.45 (d, *J* = 7.8 Hz, 1H), 7.31 (s, 1H), 7.27–7.22 (m, 2H), 7.17 (d, *J* = 2.0 Hz, 1H), 4.37–4.30 (m, 2H), 4.23–4.13 (m, 3H), 4.02 (t, *J* = 3.4 Hz, 1H), 3.97–3.91 (m, 1H), 3.83–3.76 (m, 2H), 3.67 (s, 3H), 3.63–3.51 (m, 4H), 3.50–3.44 (m, 1H), 3.33 (dd, *J* = 12.0, 2.9 Hz, 1H), 2.65 (dt, *J* = 13.6, 8.2 Hz, 1H), 2.46–2.37 (m, 1H), 2.31–2.03 (m, 6H), 2.00 (br s, 1H), 1.95–1.54 (m, 26H), 1.52 (s, 9H), 1.51–1.42 (m, 3H), 1.01–0.82 (m, 24H). ^13^C NMR (125 MHz, CDCl_3_) δ: 175.6, 175.2, 174.5, 174.4, 174.1, 173.1, 173.0, 171.1, 155.8, 81.6, 78.0, 71.8, 69.7, 69.4, 66.7, 65.7, 60.5, 56.2, 54.8, 54.2, 54.0, 52.7, 52.2, 52.0, 39.7, 39.6, 39.4, 39.1, 38.3, 37.3, 36.7, 35.6, 35.4, 29.1, 28.2 (3C), 27.0, 26.9, 25.4, 25.1, 25.0, 24.8, 24.7, 24.54, 24.52 (2C), 24.4, 23.5, 23.4, 23.0, 22.8, 21.14, 21.05, 21.0, 20.9. IR (CDCl_3_): 3321, 2959, 1732, 1661, 1530 cm^−1^. HRMS (ESI) *m*/*z*: [M + Na]^+^ calcd for C_56_H_96_N_8_O_13_Na, 1111.6995; found, 1111.7016.

*Stapled H-l-Hyp-l-Ser-[(l-Leu)_2_-Ac_5_c]_2_-OMe* (**C’**): To a solution of Boc-protected peptide **13** (11.5 mg, 0.0110 mmol) in CH_2_Cl_2_ (1 mL) was added trifluoroacetic acid (0.0110 mL) dropwise at room temperature, and the reaction mixture was stirred for 2 days at the same temperature. The reaction mixture was neutralized by adding sat. aq NaHCO_3_, and the aqueous phase was extracted with CHCl_3_ three times. The combined organic extracts were dried over anhydrous MgSO_4_ and concentrated under a vacuum to give crude product **C’** (11.6 mg, quant), which was used for the next step without further purification. *R*_f_ = 0.20 (EtOAc). Mp 105–107 °C. ${\left[\alpha \right]}_{D}^{27}$ −8.7 (*c* 1.00, CHCl_3_). ^1^H NMR (500 MHz, CDCl_3_) δ: 8.27 (d, *J* = 5.1 Hz, 1H), 7.44 (d, *J* = 7.8 Hz, 1H), 7.33 (d, *J* = 4.6 Hz, 1H), 7.26–7.21 (m, 3H), 7.00 (d, *J* = 4.2 Hz, 1H), 4.37–4.27 (m, 2H), 4.19 (dd, *J* = 10.8, 5.9 Hz, 1H), 4.13–4.04 (m, 2H), 3.97–3.86 (m, 2H), 3.84 (dd, *J* = 10.5, 5.9 Hz, 1H), 3.70 (dd, *J* = 10.6, 2.8 Hz, 1H), 3.67 (s, 3H), 3.64–3.59 (m, 1H), 3.59–3.53 (m, 1H), 3.48 (t, *J* = 8.9 Hz, 1H), 3.43–3.37 (m, 1H), 3.35 (d, *J* = 10.5 Hz, 1H), 3.00 (dd, *J* = 10.5, 2.9 Hz, 1H), 2.69–2.60 (m, 1H), 2.37–2.03 (m, 7H), 1.97–1.49 (m, 30H), 1.03–0.78 (m, 24H). ^13^C NMR (125 MHz, CDCl_3_) δ: 175.7, 175.3, 174.2, 174.0, 173.23, 173.20 (2C), 172.0, 79.5, 71.5, 69.3, 69.0, 66.8, 65.7, 59.6, 55.9, 54.8, 54.1 (2C), 52.4, 52.2, 51.0, 39.6, 39.5, 39.4, 39.2, 38.3, 37.2, 36.7, 36.0, 35.4, 28.9, 28.3, 27.5, 26.4, 25.23, 25.20, 25.1, 24.8, 24.6, 24.52, 24.49, 24.40, 23.5, 23.4, 23.1, 22.9, 21.3, 21.1, 21.0, 20.9. IR (KBr): 3337, 2957, 1736, 1655, 1535 cm^−1^. HRMS (ESI) *m*/*z*: [M + Na]^+^ calcd for C_51_H_88_N_8_O_11_Na, 1011.6470; found, 1011.6467.

### 3.4. Synthesis of Unstapled Peptide D and Stapled Peptide D’

*Boc-l-Ser^OPte^-(l-Leu)_2_-Ac_5_c-OMe* (**14**): To a solution of Boc-l-Ser^OPte^-OH **8** [51] (200 mg, 0.732 mmol) in CH_2_Cl_2_ (2.5 mL) were added EDCI·HCl (140 mg, 0.732 mmol) and HOBt·H_2_O (135 g, 0.878 mmol) at 0 °C, and the solution was stirred for 30 min. Then, a solution of H-(l-Leu)_2_-Ac_5_c-OMe [44] (270 mg, 0.732 mmol) in CH_2_Cl_2_ (2.5 mL) was added to the reaction mixture at the same temperature, and the resultant mixture was gradually warmed to room temperature. After stirring overnight, the CH_2_Cl_2_ was removed and the residue was diluted with EtOAc. The solution was washed successively with 1 M of HCl, water, sat. aq NaHCO_3_, and brine. The organic layer was dried over anhydrous MgSO_4_ and concentrated in vacuo to give a crude product, which was purified by flash column chromatography on silica gel (50% EtOAc in *n*-hexane) to give **14** (307 mg, 67%) as a white solid. *R*_f_ = 0.52 (60% EtOAc in *n*-hexane). Mp 109–115 °C. ${\left[\alpha \right]}_{D}^{26}$ −40.4 (*c* 0.995, CHCl_3_). ^1^H NMR (500 MHz, CDCl_3_) δ: 6.84 (br s, 2H), 6.57 (d, *J* = 4.9 Hz, 1H), 5.79 (ddt, *J* = 17.0, 10.3, 6.6 Hz, 1H), 5.38 (br s, 1H), 5.06–4.95 (m, 2H), 4.44 (td, *J* = 9.2, 4.9 Hz, 1H), 4.37–4.29 (m, 1H), 4.12 (q, *J* = 4.9 Hz, 1H), 3.76 (dd, *J* = 9.5, 4.6 Hz, 1H), 3.68 (s, 3H), 3.62 (dd, *J* = 9.2, 5.3 Hz, 1H), 3.53–3.44 (m, 2H), 2.25 (dt, *J* = 13.2, 7.7 Hz, 1H), 2.17 (dt, *J* = 13.1, 7.8 Hz, 1H), 2.10 (q, *J* = 7.3 Hz, 2H), 2.07–1.95 (m, 2H), 1.87–1.63 (m, 9H), 1.63–1.49 (m, 3H), 1.47 (s, 9H), 1.00–0.85 (m, 12H). ^13^C NMR (125 MHz, CDCl_3_) δ: 174.6, 171.6, 171.5, 171.1, 156.3, 137.8, 115.1, 81.1, 71.0, 69.4, 65.9, 55.6, 53.0, 52.3, 51.5, 40.4, 39.9, 37.2, 36.9, 30.2, 28.6, 28.2 (3C), 25.1, 24.8, 24.5, 24.4, 23.1, 23.0, 21.6, 21.4. IR (KBr): 3277, 2957, 1719, 1670, 1560 cm^−1^. HRMS (ESI) *m*/*z*: [M + Na]^+^ calcd for C_32_H_56_N_4_O_8_Na, 647.3996; found, 647.3991.

*Boc-(l-Leu)_2_-l-Ser^OPte^-(l-Leu)_2_-Ac_5_c-OMe* (**16**): To a solution of Boc-protected peptide **14** (307 mg, 0.491 mmol) in CH_2_Cl_2_ (5 mL) was added trifluoroacetic acid (0.982 mL) dropwise at room temperature, and the reaction mixture was stirred overnight at the same temperature. The reaction mixture was neutralized by adding sat. aq NaHCO_3_, and the aqueous phase was extracted with CHCl_3_ four times. The combined organic extracts were dried over anhydrous MgSO_4_ and concentrated under a vacuum to give crude product **15** (295 mg, quant), which was used for the next step without further purification. *R*_f_ = 0.20 (60% EtOAc in *n*-hexane). To a solution of Boc-(l-Leu)_2_-OH (194 mg, 0.562 mmol) in CH_2_Cl_2_ (2 mL) were added EDCI·HCl (108 mg, 0.562 mmol) and HOBt·H_2_O (103 mg, 0.674 mmol) at 0 °C, and the solution was stirred for 30 min. Then, a solution of amine **15** (295 mg, 0.562 mmol) in CH_2_Cl_2_ (2 mL) was added to the reaction mixture at the same temperature, and the resultant mixture was gradually warmed to room temperature. After stirring for 5 days, the CH_2_Cl_2_ was removed and the residue was diluted with EtOAc. The solution was washed successively with 1 M of HCl, water, sat. aq NaHCO_3_, and brine. The organic layer was dried over anhydrous MgSO_4_ and concentrated in vacuo to give a crude product, which was purified by flash column chromatography on silica gel (60% EtOAc in *n*-hexane) to give **16** (300 mg, 63%) as a white solid. *R*_f_ = 0.30 (60% EtOAc in *n*-hexane). Mp 243–246 °C. ${\left[\alpha \right]}_{D}^{25}$ −43.2 (*c* 1.02, CHCl_3_). ^1^H NMR (500 MHz, CDCl_3_) δ: 7.41 (d, *J* = 4.4 Hz, 1H), 7.30 (d, *J* = 6.6 Hz, 1H), 7.13 (d, *J* = 8.1 Hz, 1H), 7.02 (s, 1H), 6.84 (d, *J* = 3.7 Hz, 1H), 5.76 (ddt, *J* = 17.1, 10.3, 6.6 Hz, 1H), 5.05–4.92 (m, 2H), 5.00 (s, 1H), 4.42–4.34 (m, 1H), 4.34–4.27 (m, 1H), 4.25–4.20 (m, 1H), 4.06–3.96 (m, 2H), 3.86 (dd, *J* = 10.0, 5.4 Hz, 1H), 3.67 (s, 3H), 3.67–3.64 (m, 1H), 3.50–3.38 (m, 2H), 2.27–2.00 (m, 6H), 1.87–1.51 (m, 18H), 1.48 (s, 9H), 1.02–0.83 (m, 24H). ^13^C NMR (125 MHz, CDCl_3_) δ: 174.9, 174.3, 173.4, 172.47, 172.45, 170.9, 156.6, 137.8, 114.9, 81.5, 70.5, 68.7, 65.7, 56.3, 54.3, 54.0, 53.4, 52.2, 52.0, 40.2, 39.62 (2C), 39.59, 39.4, 37.2, 36.8, 30.1, 28.7, 28.22 (3C), 28.17 (2C), 25.0, 24.9, 24.8, 24.7, 24.4, 24.3, 23.4, 22.9, 21.5, 21.2, 20.7. IR (KBr): 3277, 2957, 1719, 1630, 1560 cm^−1^. HRMS (ESI) *m*/*z*: [M + Na]^+^ calcd for C_44_H_78_N_6_O_10_Na, 873.5677; found, 873.5658.

*Cbz-l-Pro^αAll^-(l-Leu)_2_-l-Ser^OPte^-(l-Leu)_2_-Ac_5_c-OMe* (**19**): To a solution of Boc-protected peptide **16** (112 mg, 0.132 mmol) in CH_2_Cl_2_ (1.3 mL) was added trifluoroacetic acid (0.264 mL) dropwise at room temperature, and the reaction mixture was stirred for 2 days at the same temperature. The reaction mixture was neutralized by adding sat. aq NaHCO_3_, and the aqueous phase was extracted with CHCl_3_ three times. The combined organic extracts were dried over anhydrous MgSO_4_ and concentrated under a vacuum to give crude product **17** (85.8 mg, 87%), which was used for the next step without further purification. *R*_f_ = 0.37 (EtOAc). To a solution of Cbz-l-Pro^αAll^-OH (**18** [52,53]; 27.2 mg, 0.0939 mmol) in CH_2_Cl_2_ (1 mL) were added EDCI·HCl (18.0 mg, 0.0939 mmol) and HOBt·H_2_O (17.3 mg, 0.113 mmol) at 0 °C, and the solution was stirred for 30 min. Then, a solution of amine **17** (70.5 mg, 0.0939 mmol) in CH_2_Cl_2_ (1 mL) was added to the reaction mixture at the same temperature, and the resultant mixture was gradually warmed to room temperature. After stirring for 23 h, the CH_2_Cl_2_ was removed and the residue was diluted with EtOAc. The solution was washed successively with 1 M of HCl, water, sat. aq NaHCO_3_, and brine. The organic layer was dried over anhydrous MgSO_4_ and concentrated in vacuo to give a crude product, which was purified by flash column chromatography on silica gel (50% EtOAc in *n*-hexane) to give **19** (77.0 mg, 80%) as a colorless oil. *R*_f_ = 0.60 (60% EtOAc in *n*-hexane). ${\left[\alpha \right]}_{D}^{25}$ −8.6 (*c* 2.13, CHCl_3_). ^1^H NMR (500 MHz, CDCl_3_) δ: 7.65 (br s, 1H), 7.51 (br s, 1H), 7.42–7.35 (m, 3H), 7.35–7.31 (m, 2H), 7.29 (d, *J* = 6.6 Hz, 1H), 7.19 (d, *J* = 7.8 Hz, 1H), 7.08 (s, 1H), 6.33 (br s, 1H), 5.82–5.63 (m, 2H), 5.18 (s, 2H), 5.17–5.05 (m, 2H), 5.00–4.89 (m, 2H), 4.37 (q, *J* = 7.5 Hz, 1H), 4.32–4.24 (m, 2H), 4.10–3.98 (m, 2H), 3.92–3.85 (m, 1H), 3.83–3.71 (m, 2H), 3.67 (s, 3H), 3.54–3.47 (m, 1H), 3.45 (t, *J* = 6.6 Hz, 2H), 2.96 (dd, *J* = 14.2, 7.3 Hz, 1H), 2.76 (dd, *J* = 14.2, 7.6 Hz, 1H), 2.28–2.11 (m, 5H), 2.11–1.99 (m, 5H), 1.91–1.54 (m, 17H), 1.46–1.38 (m, 1H), 1.05–0.81 (m, 24H). ^13^C NMR (125 MHz, CDCl_3_) δ: 175.3, 175.0, 174.6, 174.1, 172.62, 172.60, 171.0, 155.2, 138.1, 136.0, 132.0, 128.7 (2C), 128.5, 127.4 (2C), 120.5, 114.6, 70.2, 69.0, 68.9, 67.5, 65.7, 56.7, 54.6, 54.2, 53.5, 52.12, 52.11, 48.6, 39.7 (2C), 39.4, 39.3, 37.6, 37.1, 36.8, 35.7, 30.2, 28.7, 25.3, 25.0, 24.9, 24.8, 24.4 (2C), 24.3, 23.4 (2C), 23.3 (2C), 23.1, 22.9, 21.3, 20.9. IR (CDCl_3_): 3323, 2959, 1663, 1531 cm^−1^. HRMS (ESI) *m*/*z*: [M + Na]^+^ calcd for C_55_H_87_N_7_O_11_Na, 1044.6361; found, 1044.6360.

*H-l-Pro^αAll^-(l-Leu)_2_-l-Ser^OPte^-(l-Leu)_2_-Ac_5_c-OMe* (**D**): To a solution of Cbz-protected peptide **19** (21.4 mg, 0.0209 mmol) in MeOH (2 mL) was added 10% Pd-C (10 mg, 50 wt %) under a nitrogen atmosphere. After being vigorously stirred under a hydrogen atmosphere for 2 days at room temperature, the reaction mixture was passed through a short plug of Celite. The filtrate was concentrated under a vacuum to give a crude product, which was purified by flash column chromatography on silica gel (70% EtOAc in *n*-hexane) to give **D** (14.5 mg, 78%) as a white solid. *R*_f_ = 0.38 (EtOAc). ${\left[\alpha \right]}_{D}^{25}$ −54.3 (*c* 1.60, CHCl_3_). ^1^H NMR (500 MHz, CDCl_3_) δ: 8.45 (d, *J* = 4.4 Hz, 1H), 7.32 (d, *J* = 5.1 Hz, 1H), 7.28 (d, *J* = 8.0 Hz, 1H), 7.19–7.10 (m, 2H), 6.99 (s, 1H), 4.43–4.29 (m, 2H), 4.22–4.14 (m, 2H), 4.00–3.92 (m, 1H), 3.85 (dd, *J* = 9.9, 4.8 Hz, 1H), 3.70 (dd, *J* = 9.8, 3.4 Hz, 1H), 3.67 (s, 3H), 3.48–3.37 (m, 2H), 3.16–3.07 (m, 1H), 2.84 (dt, *J* = 10.5, 5.4 Hz, 1H), 2.27–2.12 (m, 3H), 2.12–1.94 (m, 3H), 1.86 (d, *J* = 12.5 Hz, 2H), 1.83–1.61 (m, 18H), 1.59–1.46 (m, 4H), 1.41–1.22 (m, 4H), 1.04–0.81 (m, 30H). ^13^C NMR (125 MHz, CDCl_3_) δ: 179.4, 174.9, 173.9, 173.4, 172.5, 172.4, 171.0, 71.5, 70.0, 68.6, 65.7, 56.2, 54.5, 53.3, 52.3, 52.2, 52.0, 47.4, 41.3, 40.4, 39.6, 39.3, 38.5, 37.2, 37.0, 36.8, 29.3, 28.0, 26.3, 24.9, 24.8 (3C), 24.4, 24.3, 23.6, 23.4, 23.2, 22.9, 22.5, 21.3, 21.2, 21.1, 20.7, 18.6, 14.4, 14.0. IR (CDCl_3_): 3327, 2961, 1734, 1663, 1530 cm^−1^. HRMS (ESI) *m*/*z*: [M + Na]^+^ calcd for C_47_H_85_N_7_O_9_Na, 914.6306; found, 914.6267.

*Stapled H-l-Pro-(l-Leu)_2_-l-Ser-(l-Leu)_2_-Ac_5_c-OMe* (**D’**): Under an argon atmosphere, to a solution of **19** (22.3 mg, 0.0218 mmol) in CH_2_Cl_2_ (1 mL) was added second-generation Grubbs catalyst (3.7 mg, 4.4 μmol) at room temperature, and the mixture was stirred for 2 h at the same temperature. The reaction mixture was filtered through a short pad of silica gel (EtOAc) and concentrated. The crude material was purified by flash chromatography on silica gel (80% EtOAc in *n*-hexane) to provide stapled peptide **20** (21.0 mg, 96%) as a mixture of *E*- and *Z*-isomers (*E*/*Z* = 1.1:1). *R*_f_ (*E*-form) = 0.35 (50% EtOAc in *n*-hexane), *R*_f_ (*Z*-form) = 0.26 (50% EtOAc in *n*-hexane). Next, to a solution of stapled peptides **20** (28.1 mg, 0.0283 mmol) in MeOH (1.5 mL) was added 10% Pd-C (15 mg, 50 wt %) under a nitrogen atmosphere. After being vigorously stirred under a hydrogen atmosphere for 21 h at room temperature, the reaction mixture was passed through a short plug of Celite. The filtrate was concentrated under a vacuum to give a crude product, which was purified by flash column chromatography on silica gel (5% MeOH in CHCl_3_) to give **D’** (13.6 mg, 71%) as a white solid. *R*_f_ = 0.16 (3% MeOH in CHCl_3_). ${\left[\alpha \right]}_{D}^{23}$ −30.2 (*c* 1.38, CHCl_3_). ^1^H NMR (500 MHz, CDCl_3_) δ: 8.49 (br s, 1H), 7.27 (d, *J* = 7.3 Hz, 1H), 7.23 (d, *J* = 6.1 Hz, 1H), 7.09 (d, *J* = 7.8 Hz, 1H), 6.96 (s, 1H), 6.38 (br s, 1H), 4.48 (ddd, *J* = 10.0, 6.1, 3.4 Hz, 1H), 4.40–4.27 (m, 2H), 4.03–3.93 (m, 2H), 3.90 (dd, *J* = 9.8, 3.4 Hz, 1H), 3.77–3.68 (m, 1H), 3.68 (s, 3H), 3.54 (dt, *J* = 9.2, 4.5 Hz, 1H), 3.50–3.43 (m, 1H), 3.14–3.06 (m, 1H), 2.86–2.78 (m, 1H), 2.31–2.20 (m, 2H), 2.19–2.12 (m, 1H), 2.11–1.99 (m, 2H), 1.99–1.91 (m, 1H), 1.90–1.58 (m, 21H), 1.57–1.36 (m, 7H), 1.05–0.82 (m, 24H). ^13^C NMR (125 MHz, CDCl_3_) δ: 174.9, 174.2, 173.39, 173.38, 172.5, 172.4, 170.4, 69.0, 68.8, 65.8, 56.0, 54.5, 54.3, 53.5, 52.2, 52.0, 46.6, 40.3, 40.1, 39.68, 39.66, 38.7, 37.2, 36.9, 36.8, 28.2, 27.4, 26.4, 25.5, 25.2, 25.1, 24.9, 24.7, 24.40, 24.35 (2C), 24.2, 23.3 (2C), 23.0, 22.7, 21.8, 21.2, 21.1, 21.0. IR (KBr): 3296, 3109, 2953, 1641, 1530 cm^−1^. HRMS (ESI) *m*/*z*: [M + Na]^+^ calcd for C_45_H_79_N_7_O_9_Na, 884.5837; found, 884.5874.

### 3.5. General Procedure for Peptide-Catalyzed Michael Addition of 1-Methylindole to α,β-Unsaturated Aldehyde

*Michael adduct **23**: To a mixture of (E)-4-nitrocinnamaldehyde* (**21**; 8.9 mg, 0.050 mmol), peptide catalyst (0.010 mmol), and benzoic acid (1.2 mg, 0.010 mmol) in THF (0.25 mL) was added 1-methylindole **22** (0.0187 mL, 0.150 mmol) at room temperature, and the mixture was stirred for the given time. To the reaction mixture were added NaBH_4_ (9.5 mg, 0.25 mmol) and EtOH (0.25 mL) at the same temperature, and additionally stirred for 30 min. After filtration via a short plug of silica gel (60% EtOAc in *n*-hexane), the filtrate was concentrated under a vacuum to give crude product **23** as a white solid. *R*_f_ = 0.14 (50% EtOAc in *n*-hexane). ^1^H NMR (500 MHz, CDCl_3_) δ: 8.12 (d, *J* = 8.6 Hz, 2H), 7.48 (d, *J* = 8.8 Hz, 2H), 7.36 (d, *J* = 7.8 Hz, 1H), 7.29 (d, *J* = 8.3 Hz, 1H), 7.21 (t, *J* = 7.6 Hz, 1H), 7.02 (t, *J* = 7.6 Hz, 1H), 6.96 (s, 1H), 4.55 (t, *J* = 7.7 Hz, 1H), 3.79 (s, 3H), 3.74–3.61 (m, 2H), 2.52–2.45 (m, 1H), 2.31–2.24 (m, 1H). HPLC (Chiralpak AD-H, 10% *i*-propanol in *n*-hexane, flow rate = 1.0 mL/min): *t*_R_ = 26.0 min (minor), *t*_R_ = 35.2 min (major), ee = 47%. HPLC chart is given in the Supplementary Materials.

## Figures and Tables

**Table 1:**
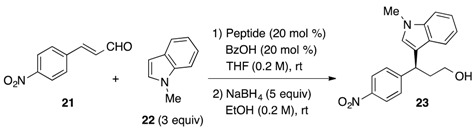
Catalytic activities of unstapled peptides **A**–**D** and stapled peptides **A’**–**D’** in the Michael addition reaction of **21** and **22**.

Entry	Peptide	Time (d)	Conv. (%) ^1^	Ee (%) ^2^
1	**A**	6	27	−1
2	**A’**	6	46	6
3	**B**	1	12	−29
4	**B’**	1	83	36
5	**C**	4	51	−11
6	**C’**	4	81	−5
7	**D**	2	50	−47
8	**D’**	2	69	−56
9	**none**	1	9	N.D. ^3^

^1^ Conversion was determined by ^1^H NMR analysis. ^2^ Ee was determined by HPLC. ^3^ Not determined.

**Table 2:**
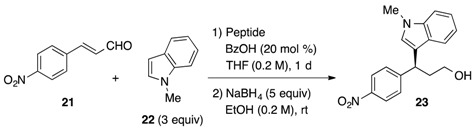
Effect of the catalyst loading and temperature in the Michael addition reaction.

Entry	Peptide (mol %)	Temp. (°C)	Conv. (%) ^1^	Ee (%) ^2^
1	**B** (20)	rt ^3^	12	−29
2	**B’** (20)	rt ^3^	83	36
3	**B** (10)	rt ^3^	6	−13
4	**B’** (10)	rt ^3^	81	36
5	**B** (5)	rt ^3^	trace	N.D. ^4^
6	**B’** (5)	rt ^3^	66	30
7	**B** (20)	0	trace	N.D. ^4^
8	**B’** (20)	0	78	47

^1^ Conversion was determined by ^1^H NMR analysis. ^2^ Ee was determined by HPLC. ^3^ Room temperature. ^4^ Not determined.

## Supplementary Materials

The following are available online, ^1^H and ^13^C NMR spectra of compounds **3**, **A**, **4**, **B**, **6**, **A’**, **7**, **B’**, **9**, **11**, **C**, **13**, **C’**, **14**, **16**, **19**, **D**, and **D’**; HPLC chart of compound **23**.
