# Supplementary material for: Design and Synthesis of Helical N-Terminal l-Prolyl Oligopeptides Possessing Hydrocarbon Stapling
Source: Molecules. 2020 Oct 13;25(20):4667. doi: 10.3390/molecules25204667 (PMC7594088; doi:10.3390/molecules25204667)
Supplement: Supplementary file 1 [file molecules-25-04667-s001.pdf]

Supporting Information

Design and synthesis of helical *N*-terminal L-prolyl oligopeptides possessing hydrocarbon stapling

Atsushi Ueda,* Mei Higuchi, Kazuki Sato, Tomohiro Umeno, Masakazu Tanaka*

Graduate School of Biomedical Sciences, Nagasaki University, 1-14 Bunkyo-machi, Nagasaki 852-8521, Japan.

E-mail for A.U.: aueda@nagasaki-u.ac.jp

E-mail for M.T.: matanaka@nagasaki-u.ac.jp

Table of Contents

1. Copies of ^{1}H and ^{13}C NMR spectra ········· S2

2. HPLC chart of compound **23** ········· S20

1. NMR spectra.

^{1}H NMR of **3** (500 MHz, $CDCl_3$)

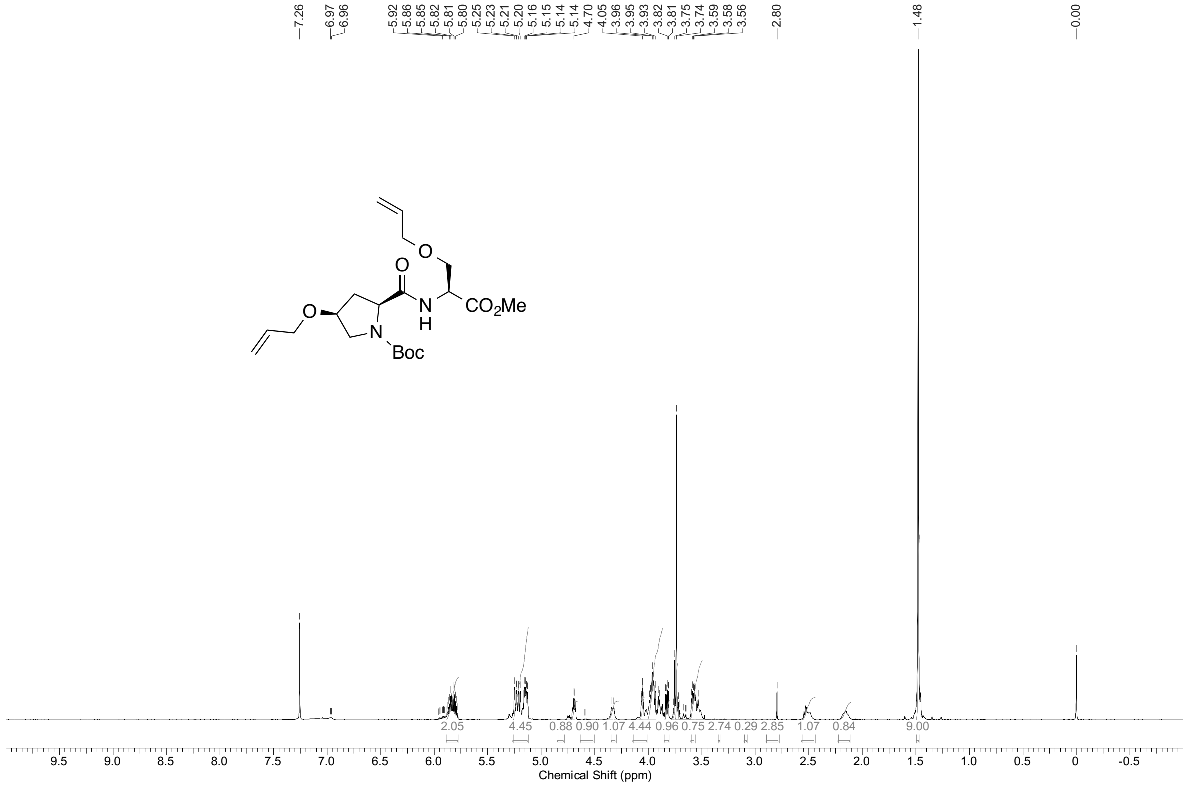

^{13}C NMR of **3** (125 MHz, $CDCl_3$)

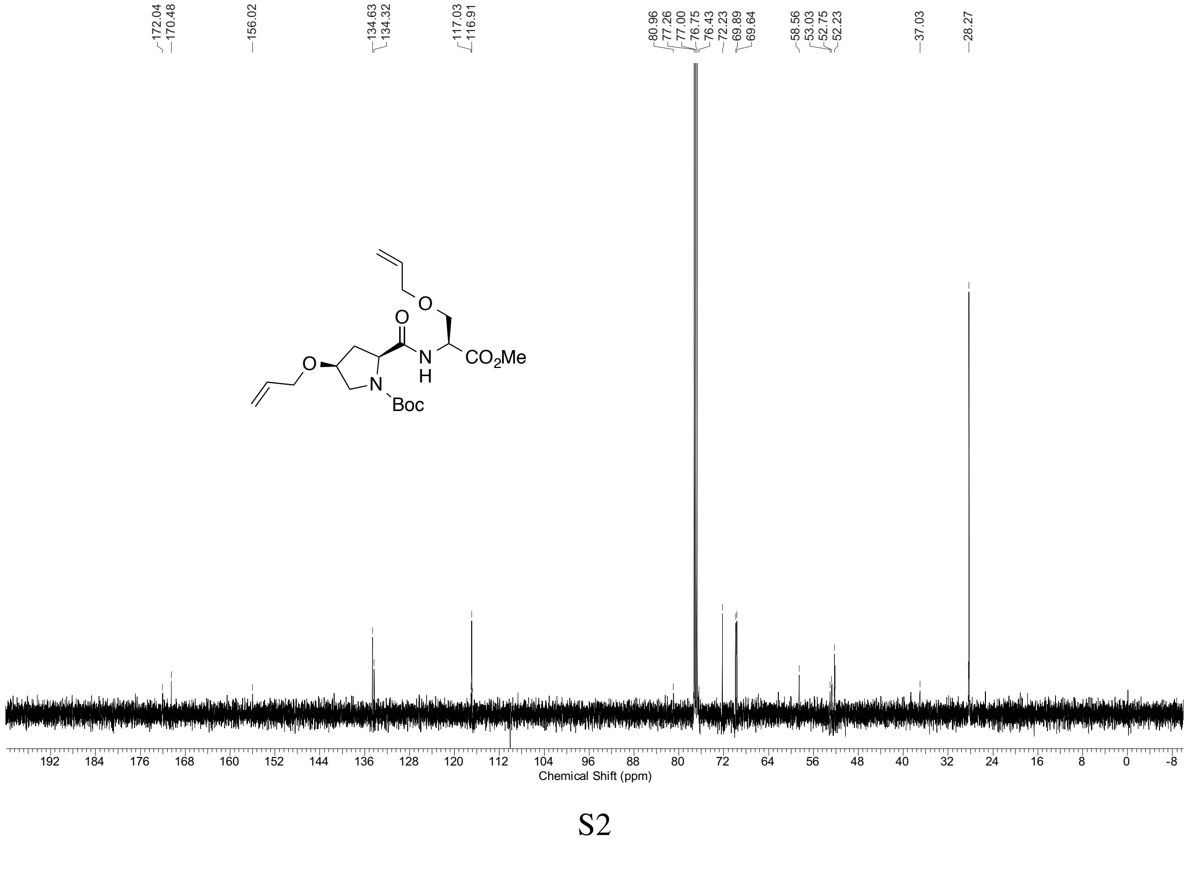

^{1}H NMR of **A** (500 MHz, $CDCl_3$)

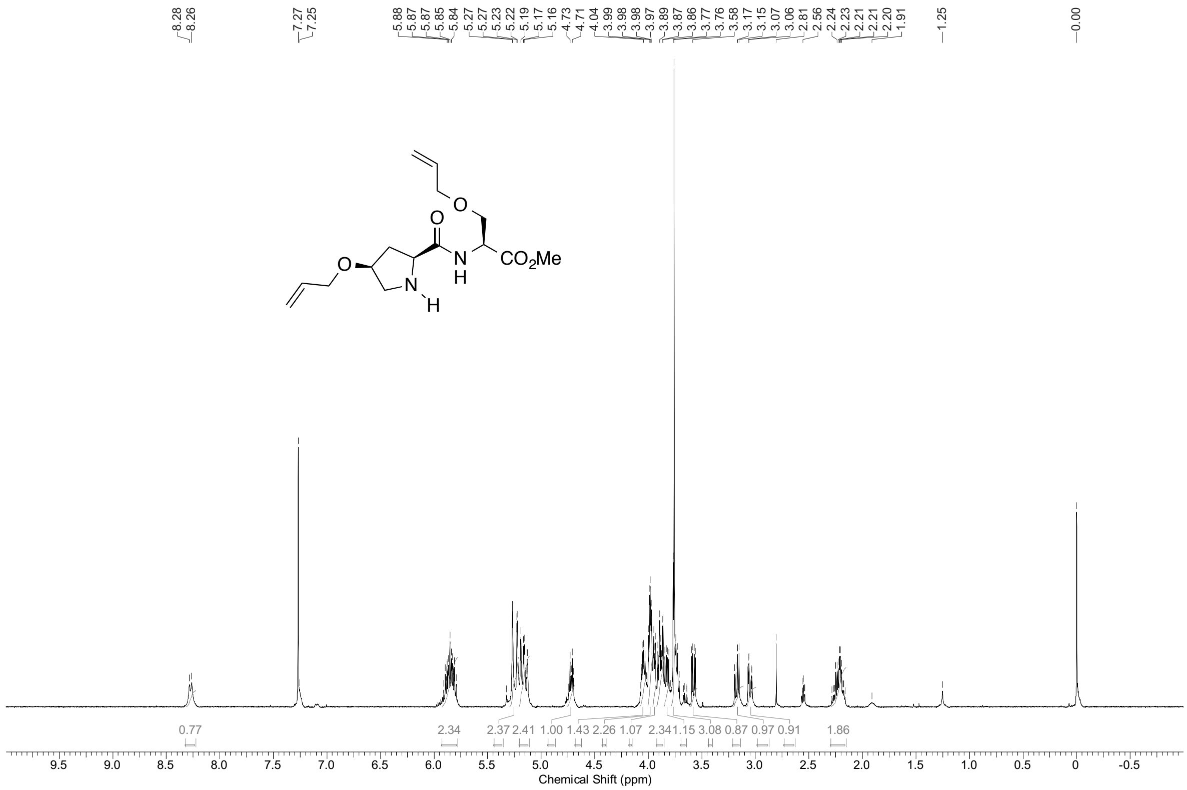

^{13}C NMR of **A** (125 MHz, $CDCl_3$)

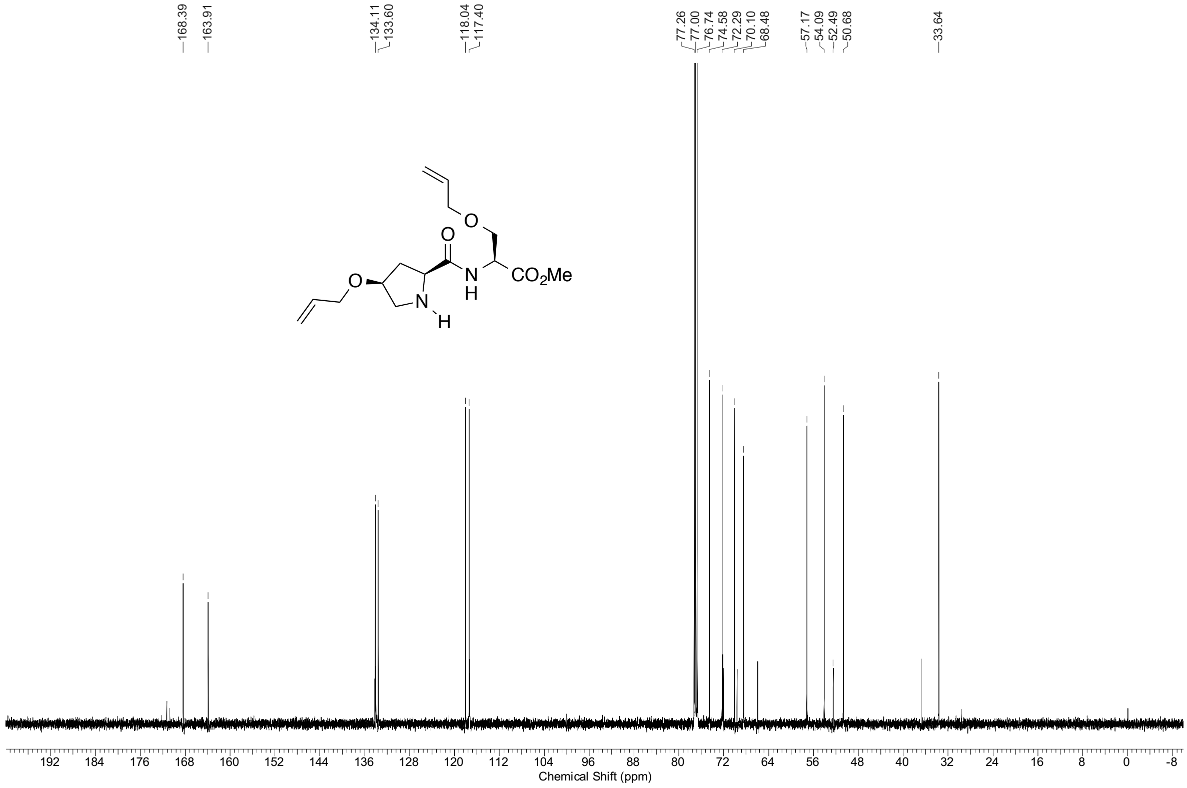

^{1}H NMR of **4** (500 MHz, $CDCl_3$)

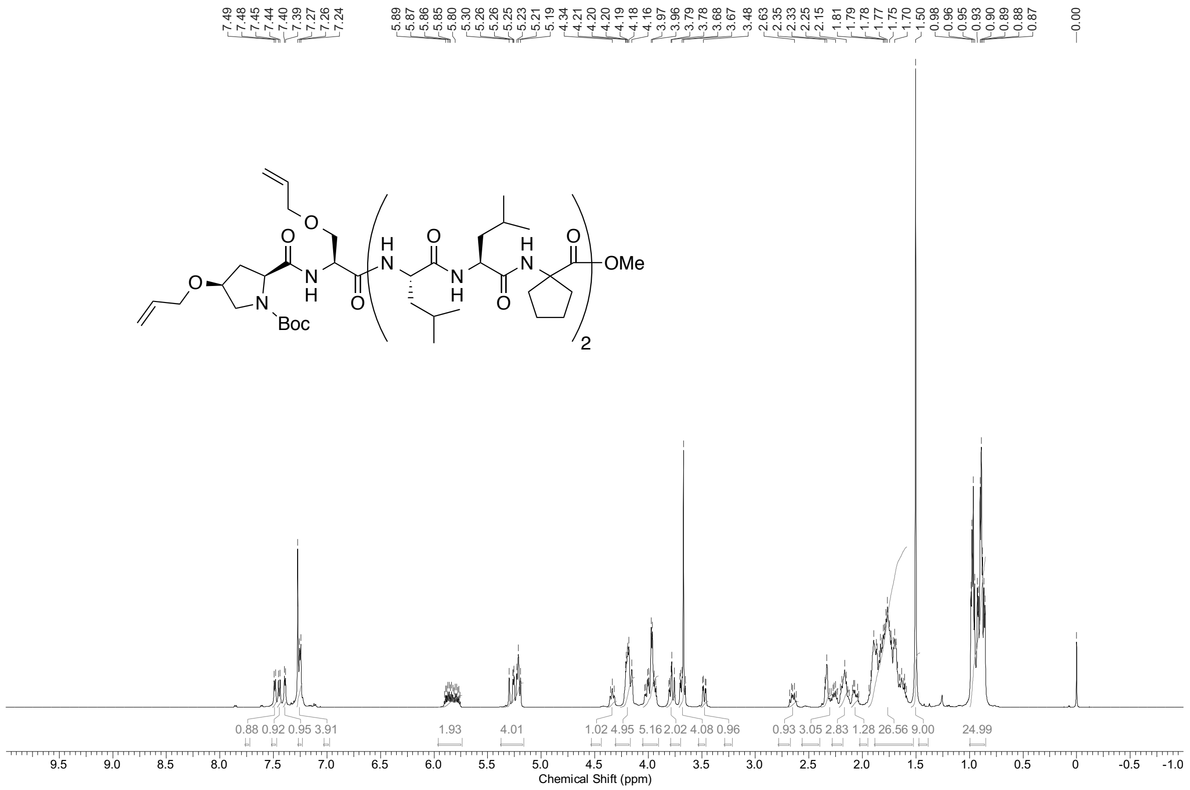

^{13}C NMR of **4** (125 MHz, $CDCl_3$)

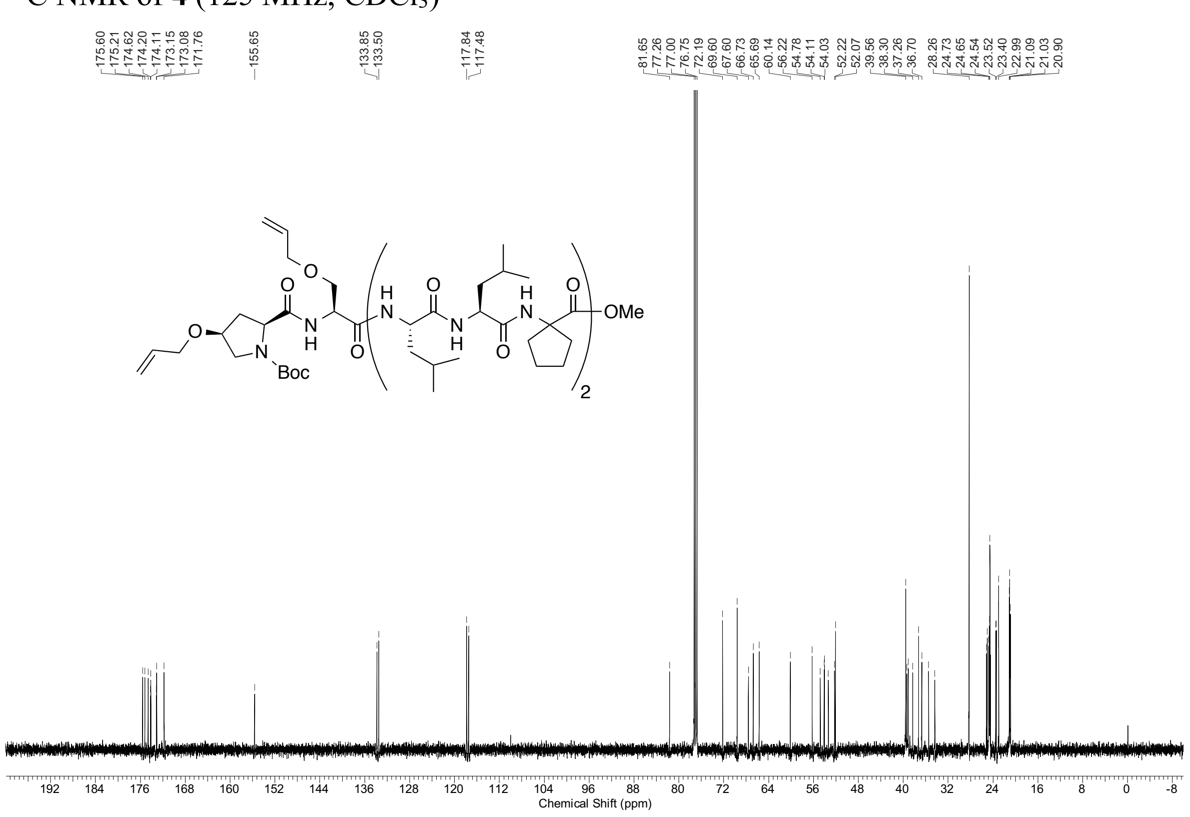

^{1}H NMR of **B** (500 MHz, $CDCl_3$)

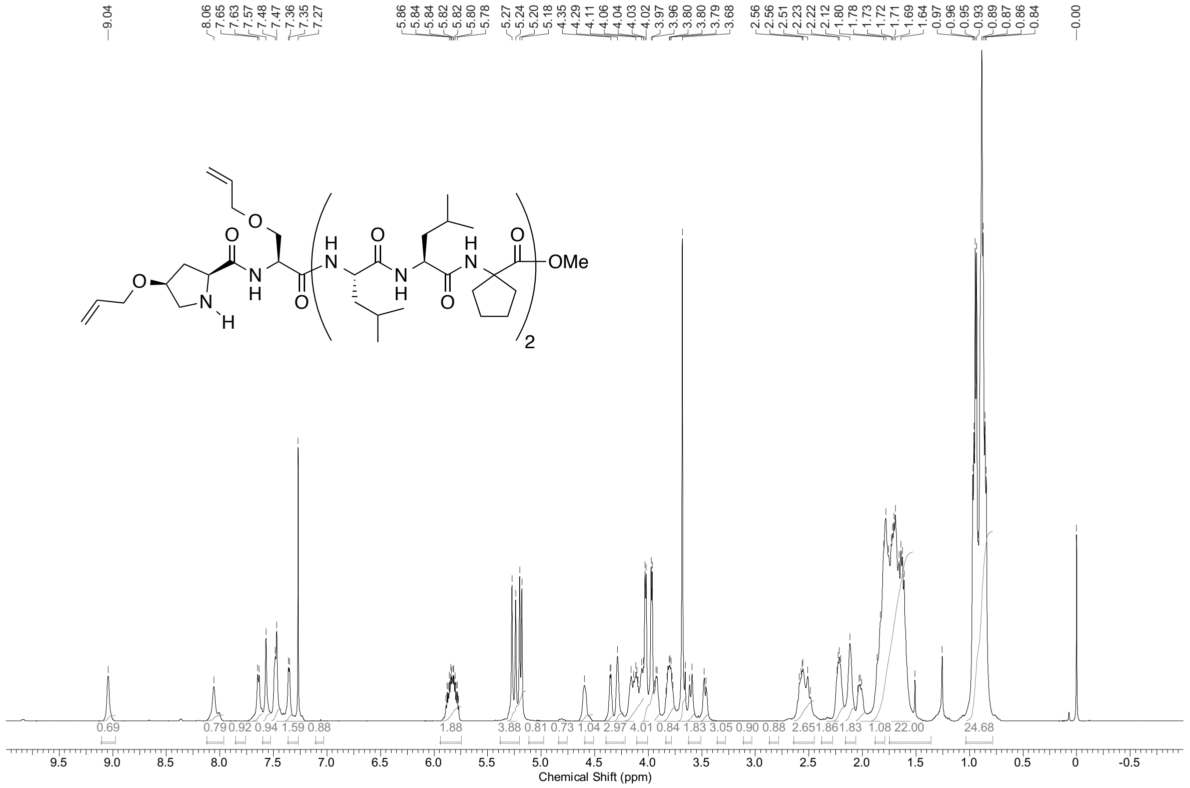

^{13}C NMR of **B** (125 MHz, $CDCl_3$)

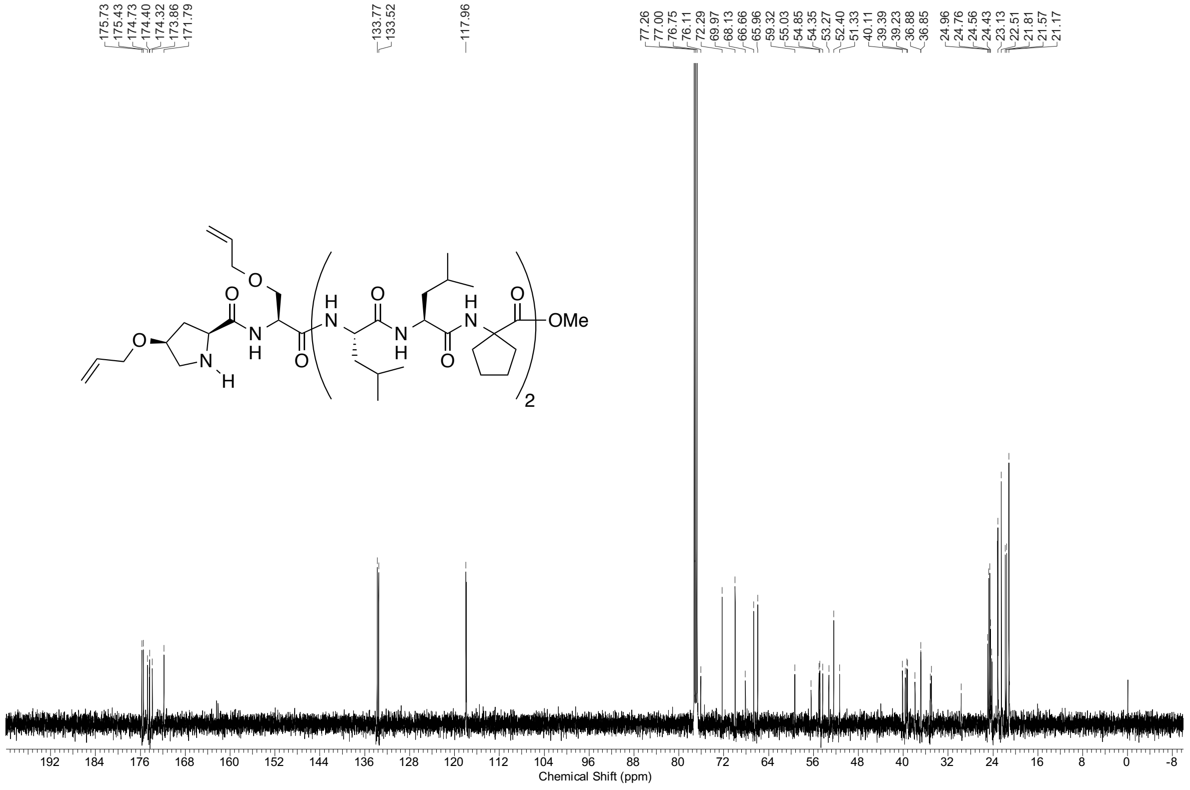

^{1}H NMR of **6** (500 MHz, $CDCl_3$)

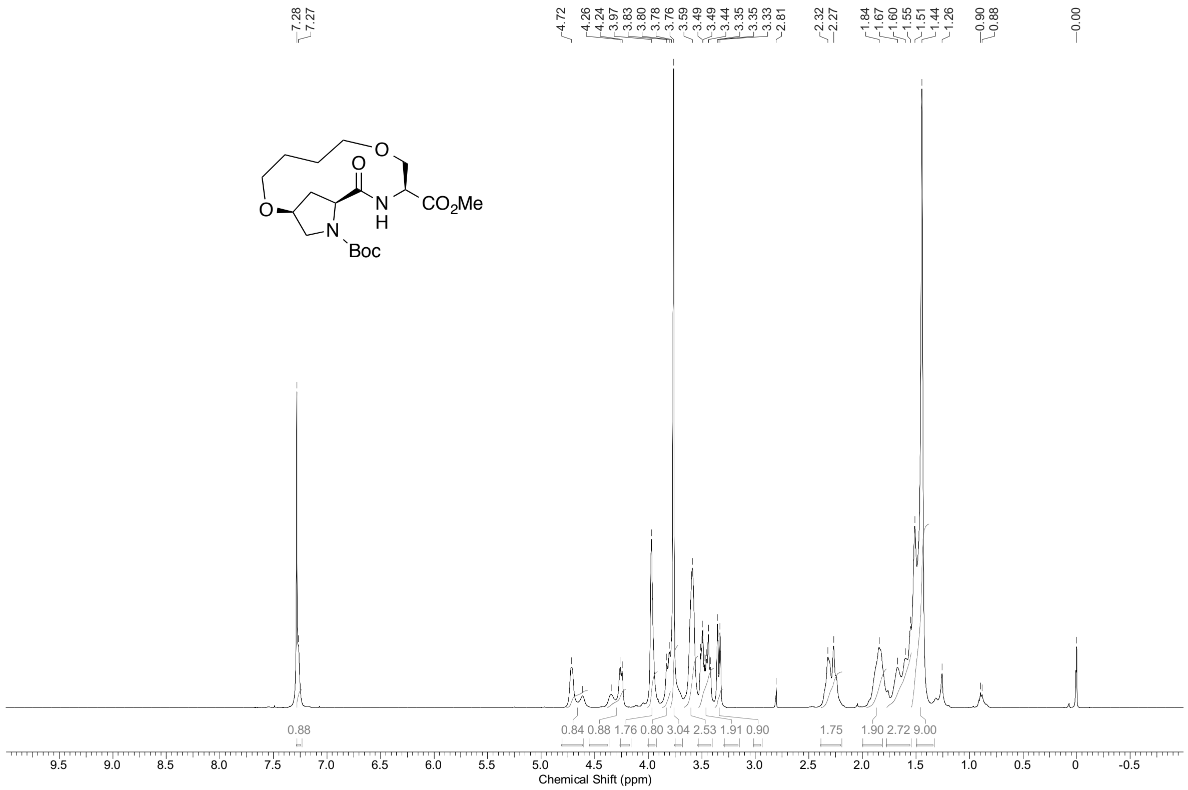

^{13}C NMR of **6** (125 MHz, $CDCl_3$)

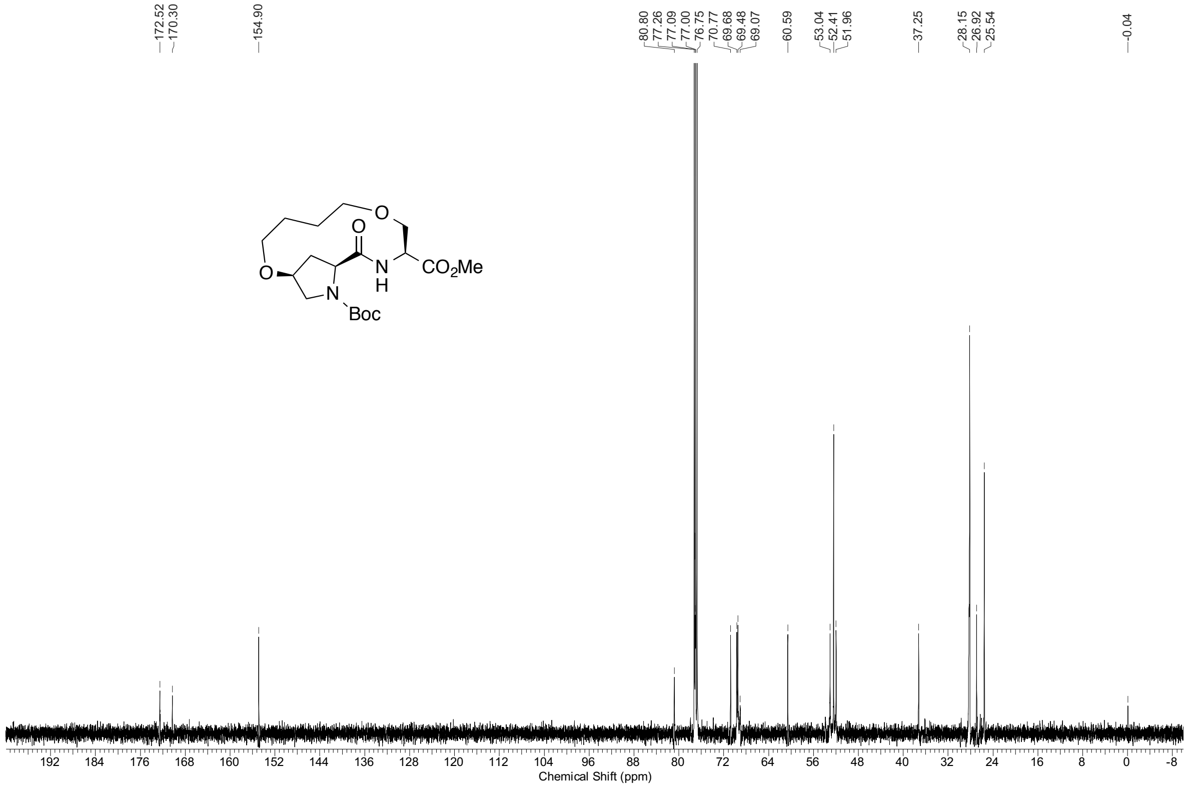

^{1}H NMR of **A’** (500 MHz, $CDCl_3$)

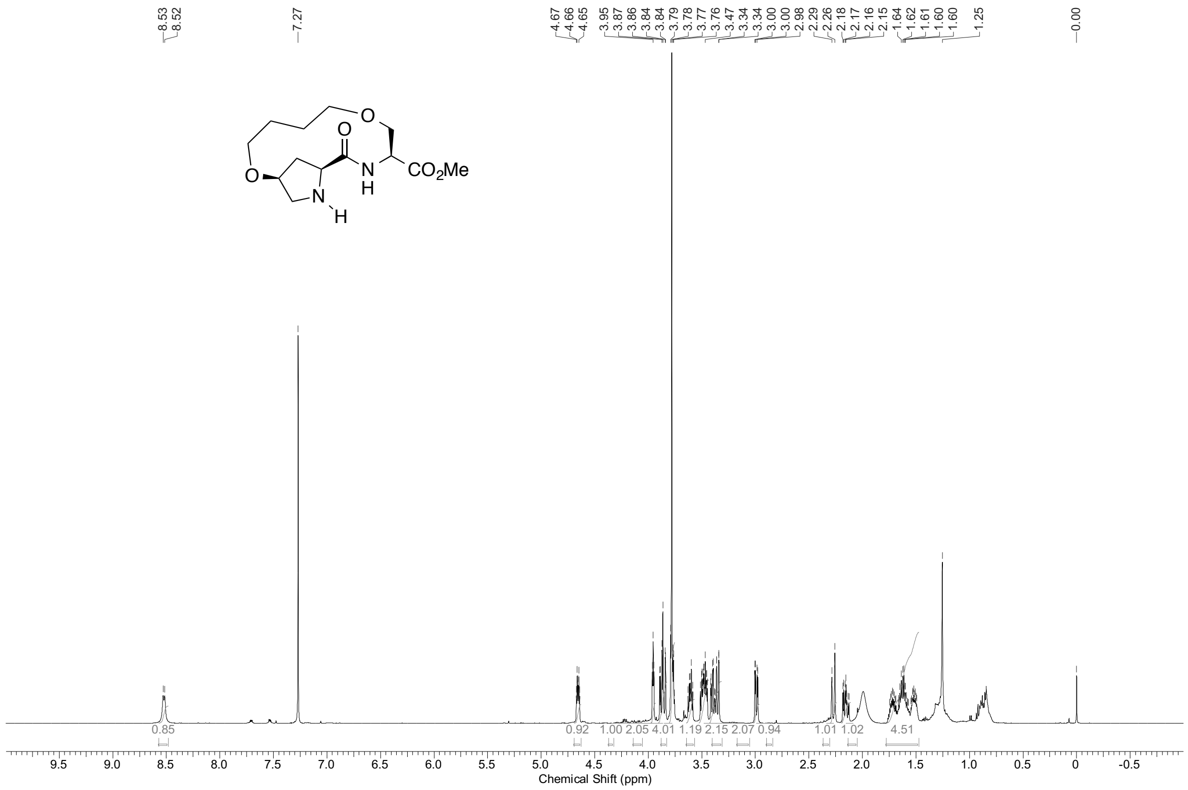

^{13}C NMR of **A’** (125 MHz, $CDCl_3$)

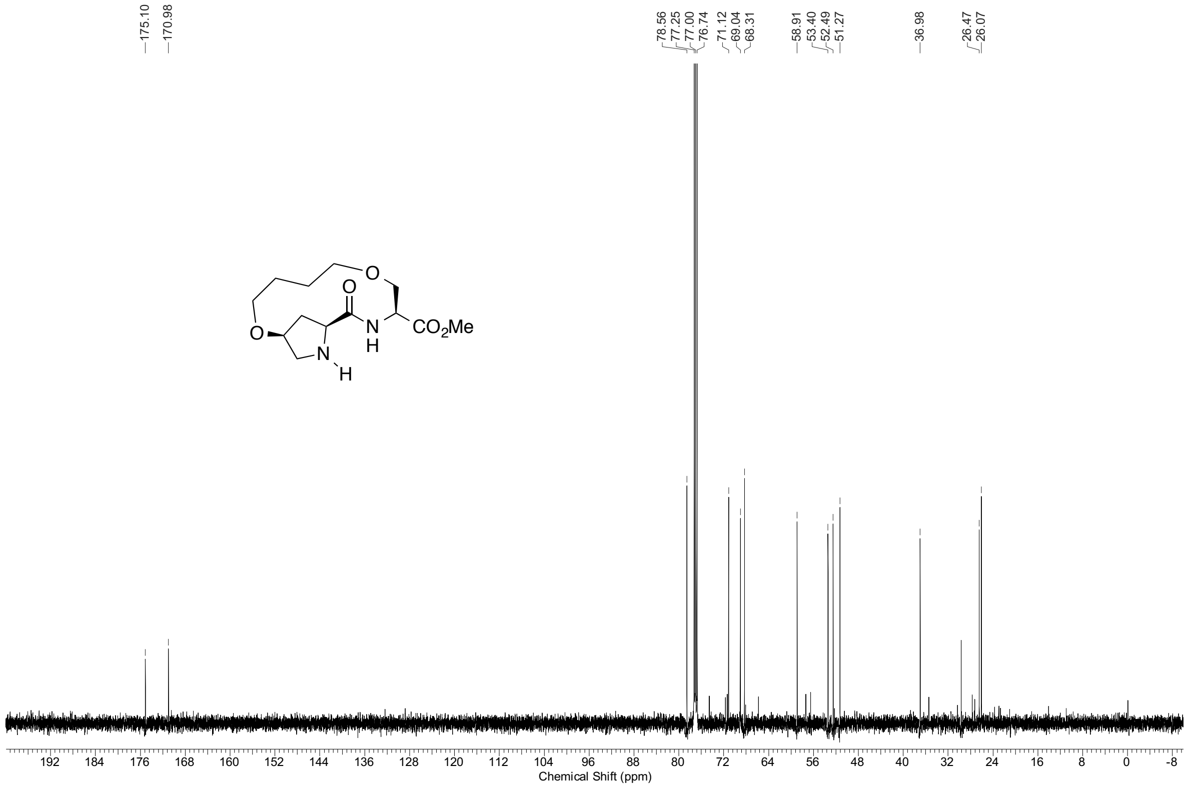

^{1}H NMR of **7** (500 MHz, $CDCl_3$)

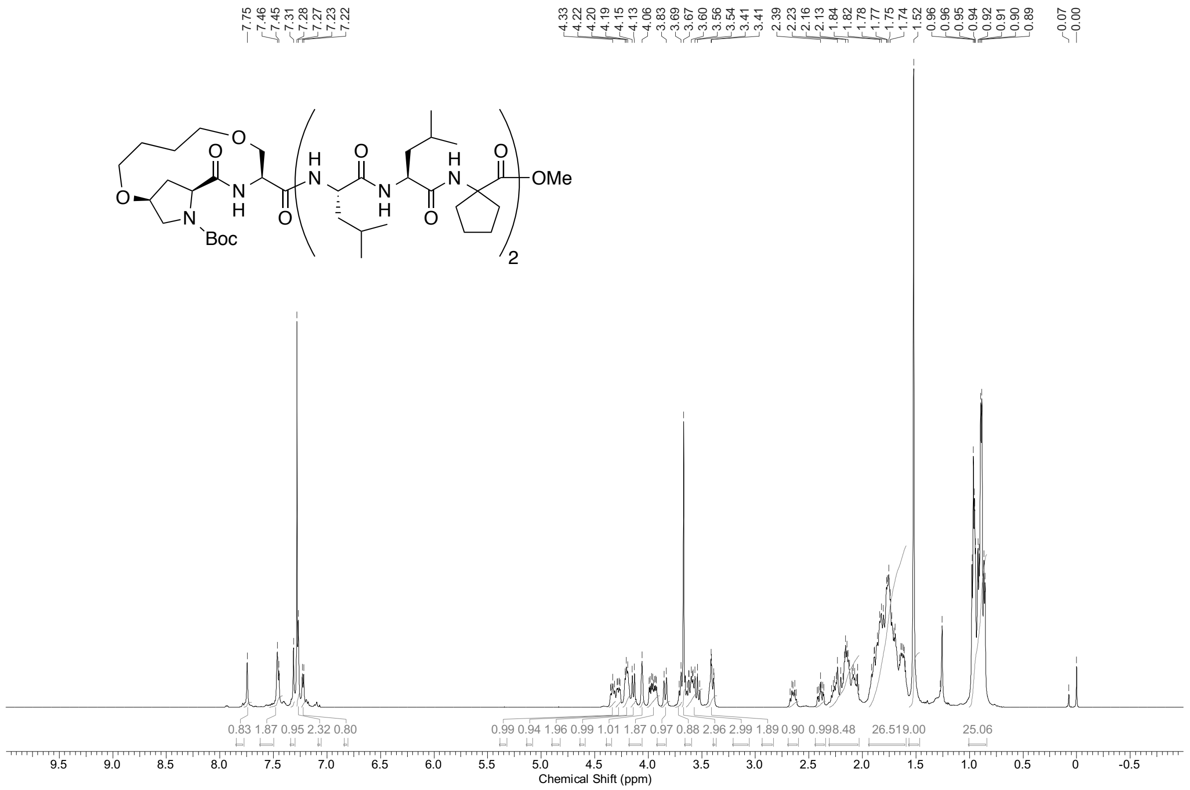

^{13}C NMR of **7** (125 MHz, $CDCl_3$)

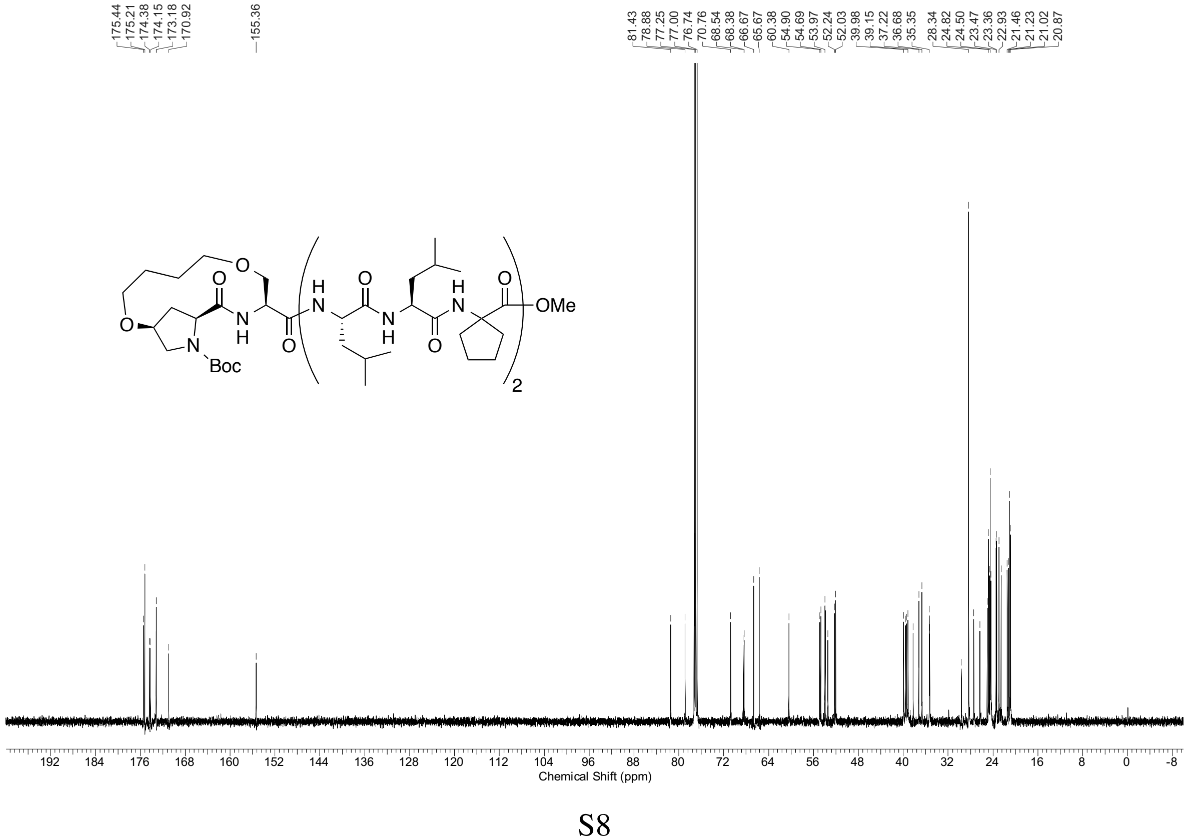

^{1}H NMR of **B’** (500 MHz, $CDCl_3$)

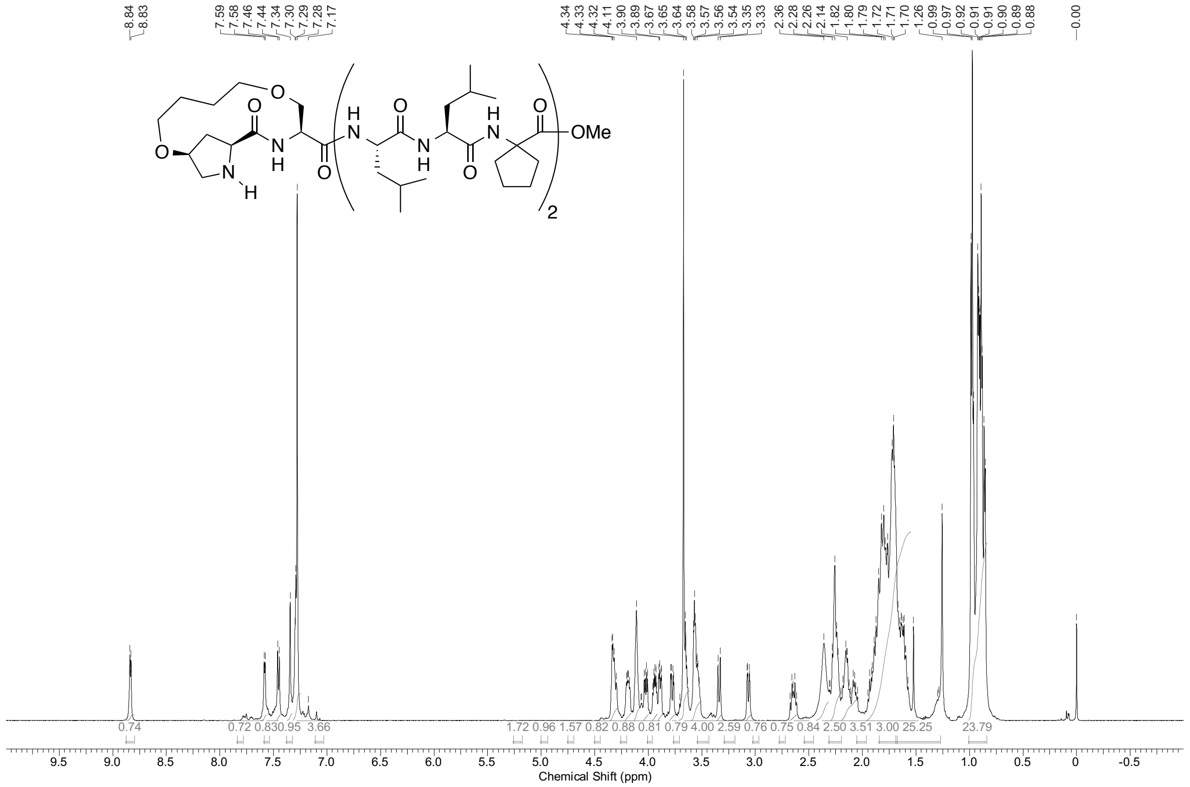

^{13}C NMR of **B’** (125 MHz, $CDCl_3$)

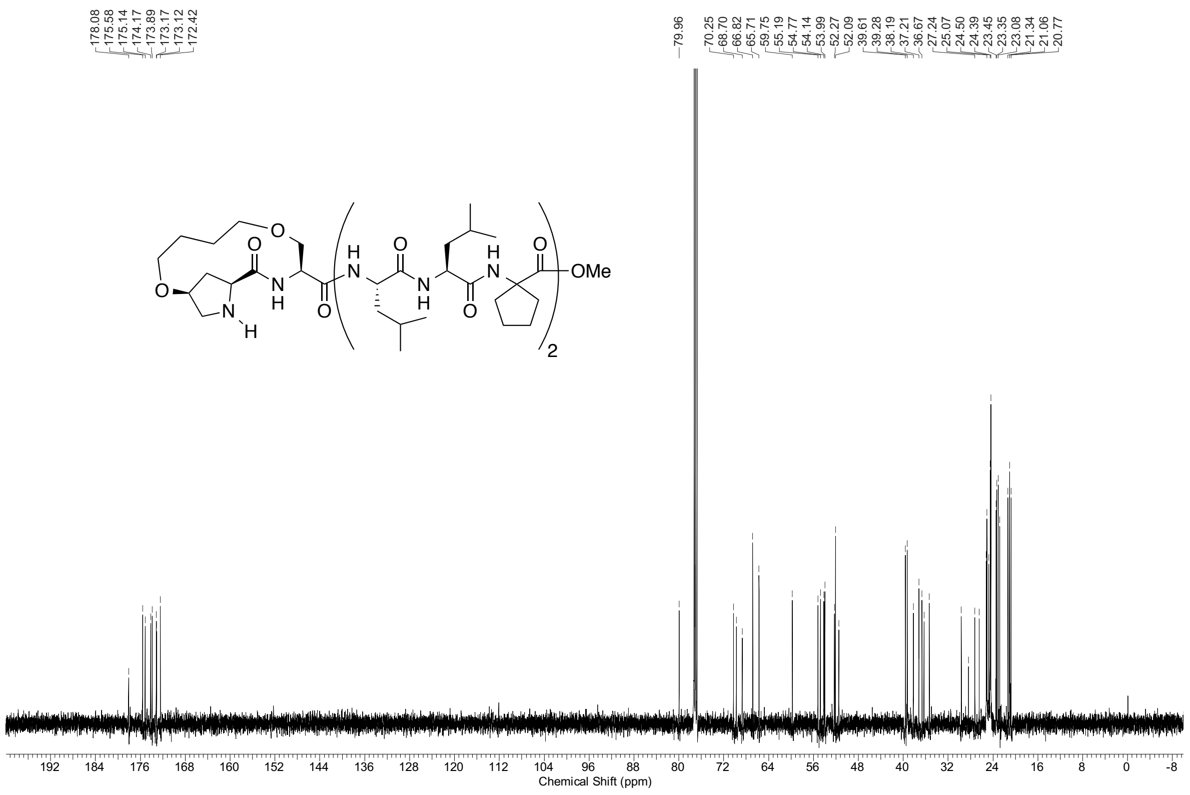

^{1}H NMR of **9** (500 MHz, $CDCl_3$)

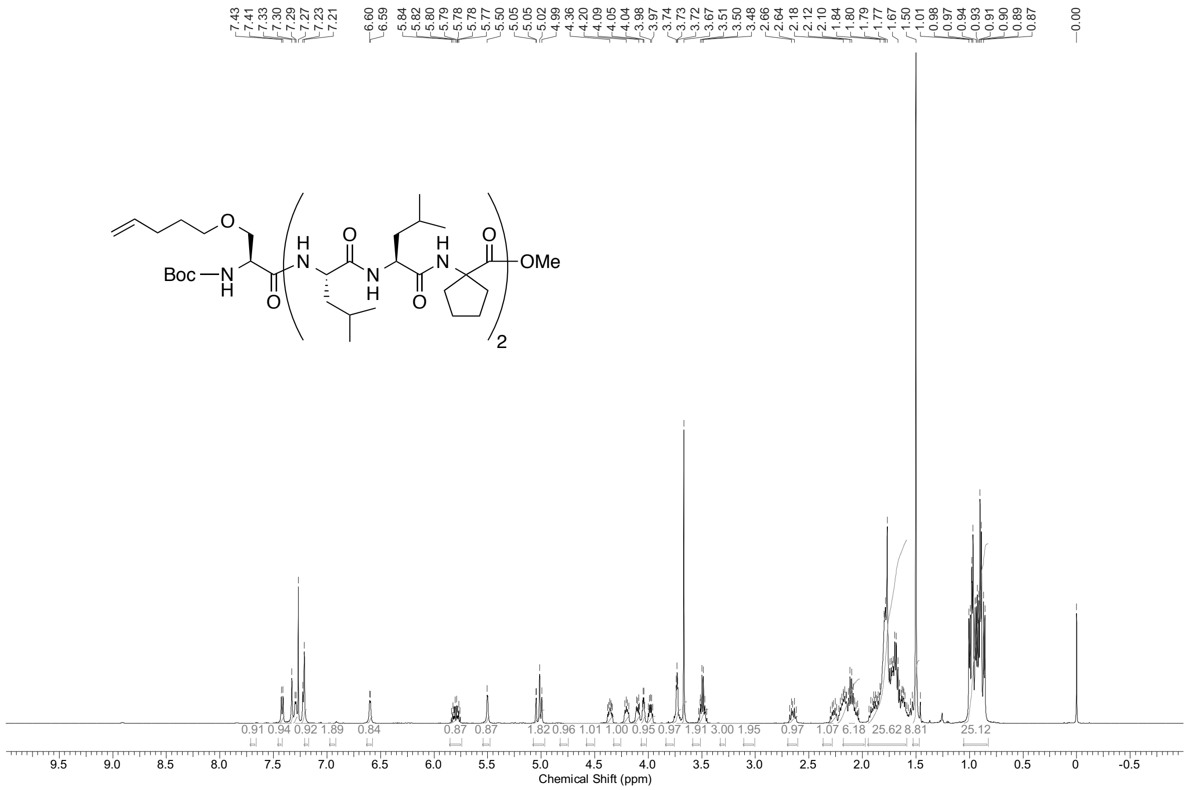

^{13}C NMR of **9** (125 MHz, $CDCl_3$)

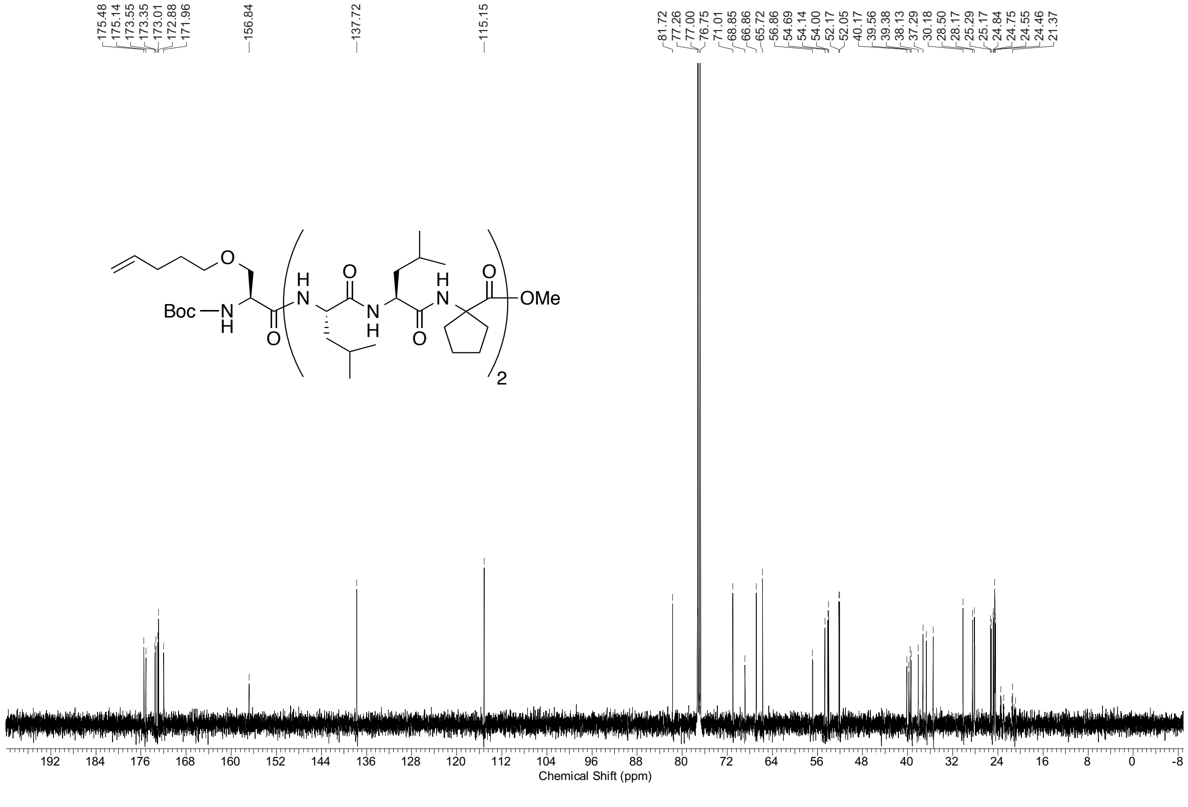

^{1}H NMR of **11** (500 MHz, $CDCl_3$)

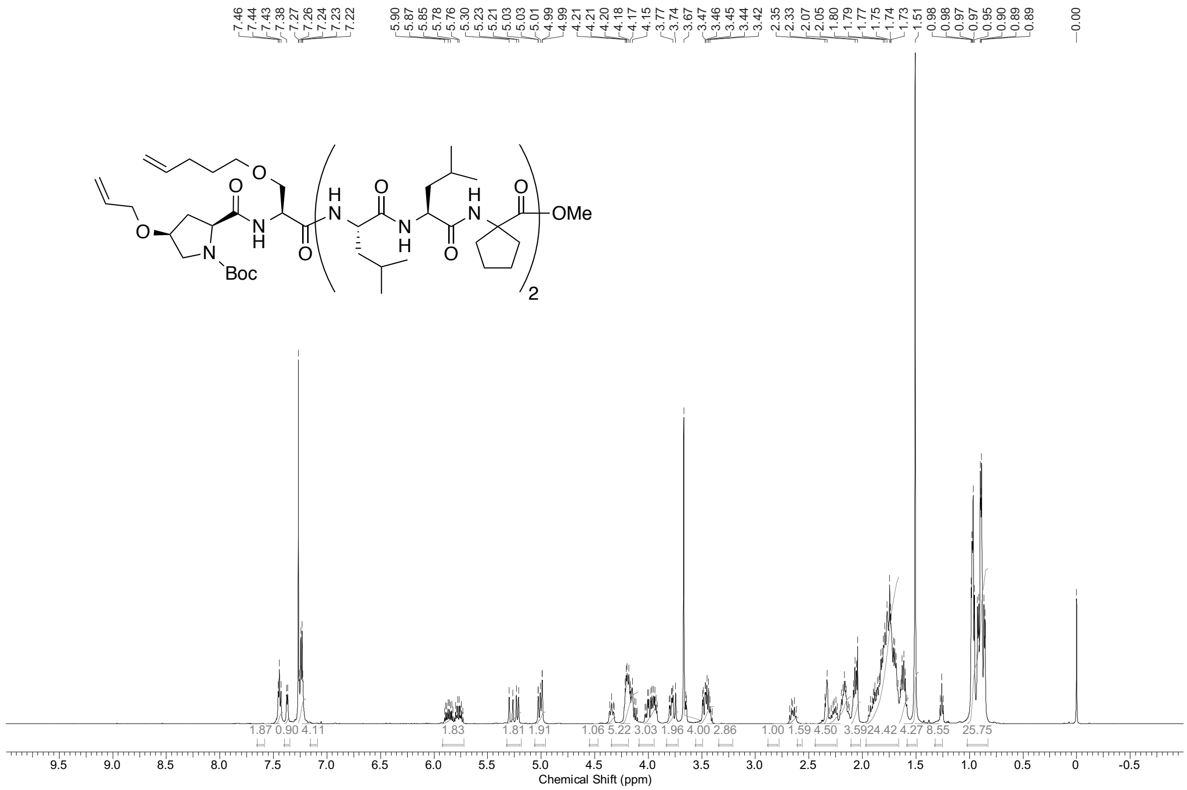

^{13}C NMR of **11** (125 MHz, $CDCl_3$)

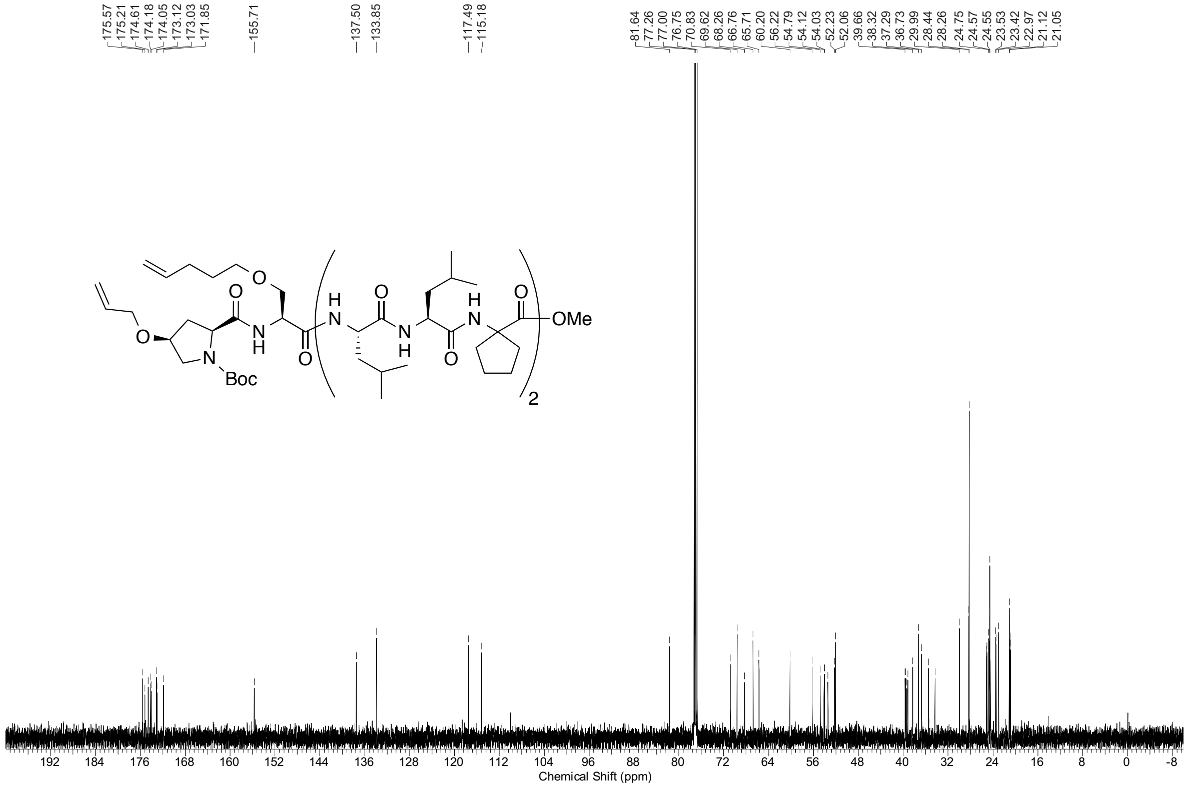

^{1}H NMR of **C** (500 MHz, $CDCl_3$)

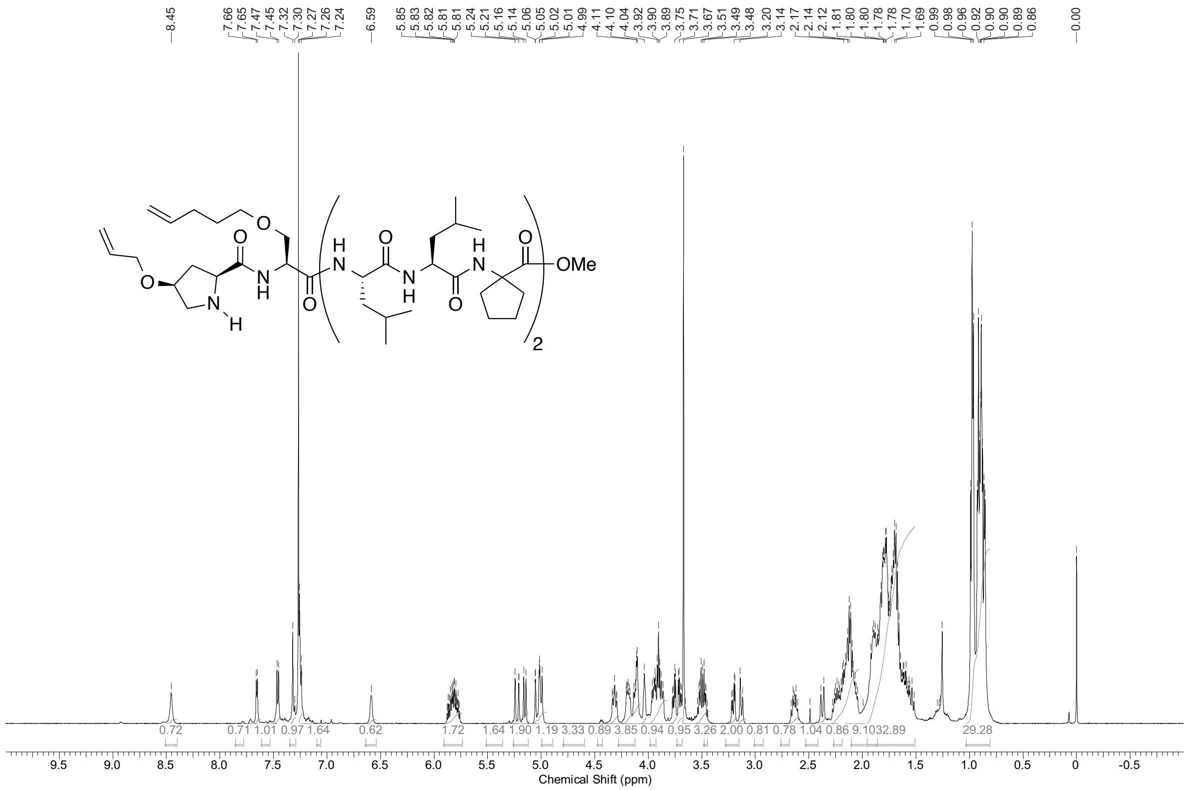

^{13}C NMR of **C** (125 MHz, $CDCl_3$)

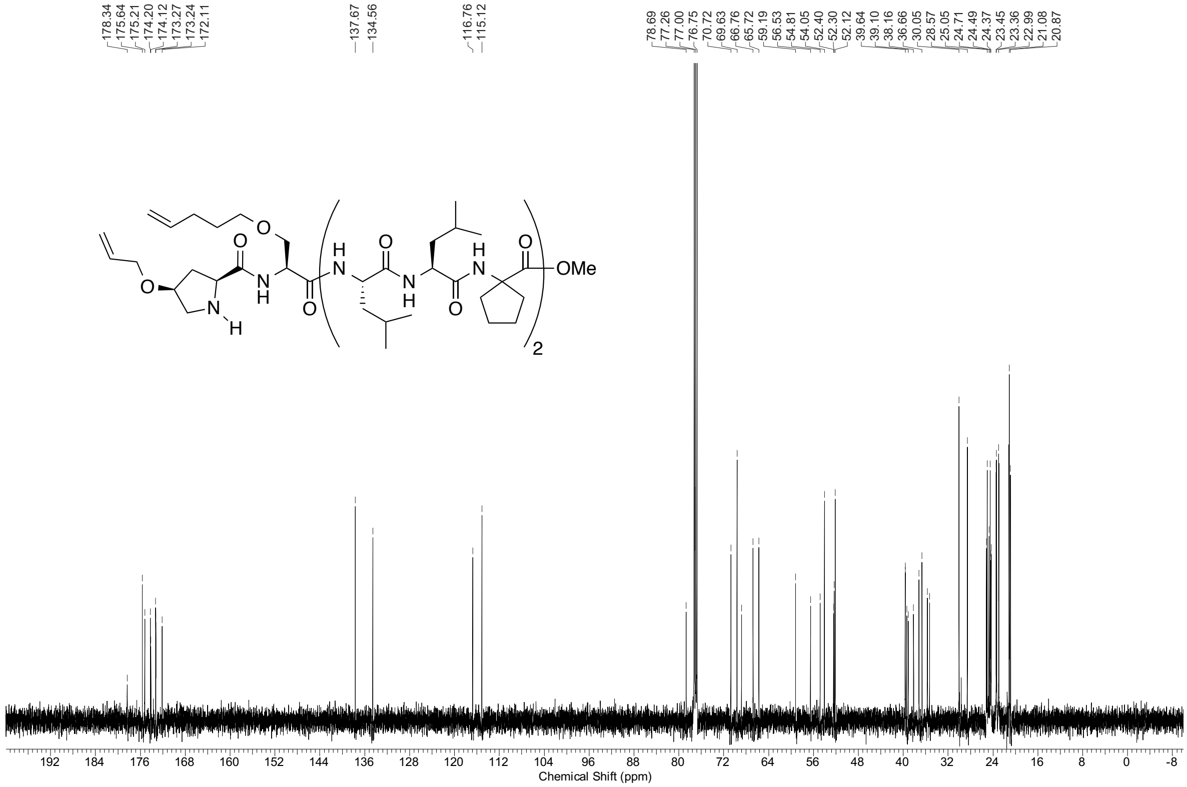

^{1}H NMR of **13** (500 MHz, $CDCl_3$)

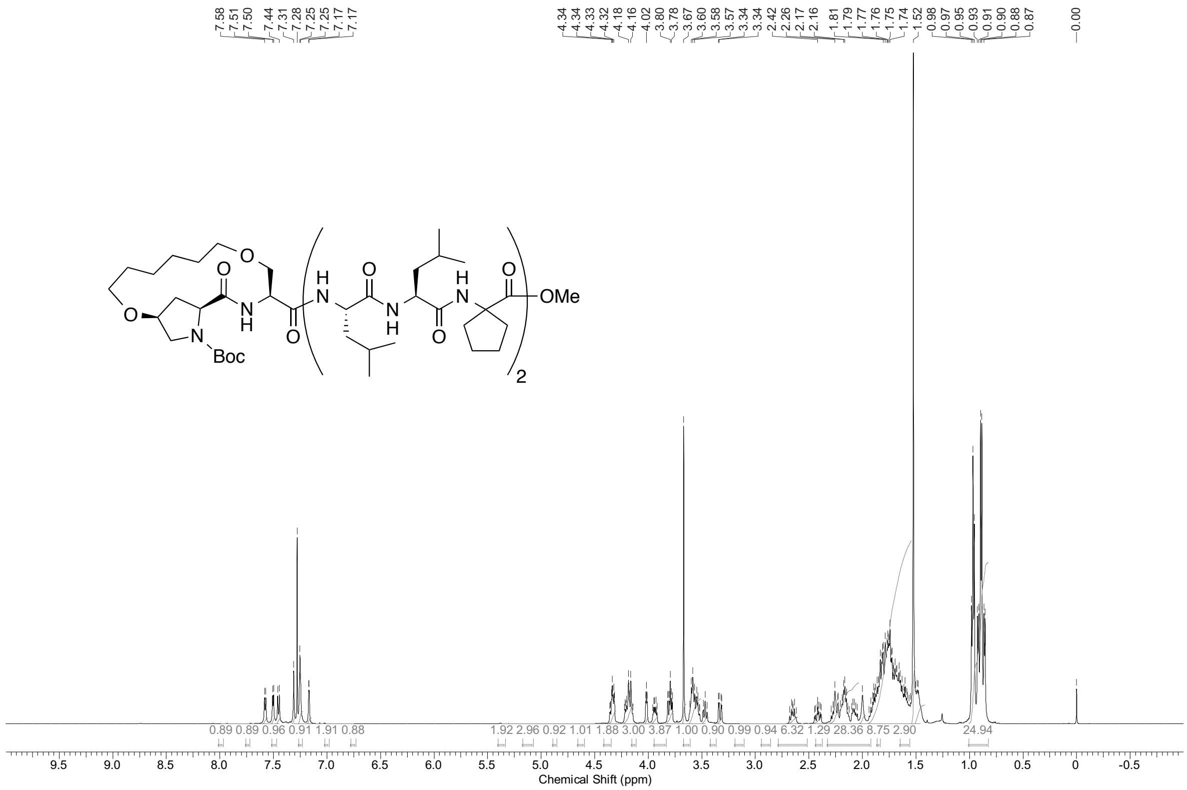

^{13}C NMR of **13** (125 MHz, $CDCl_3$)

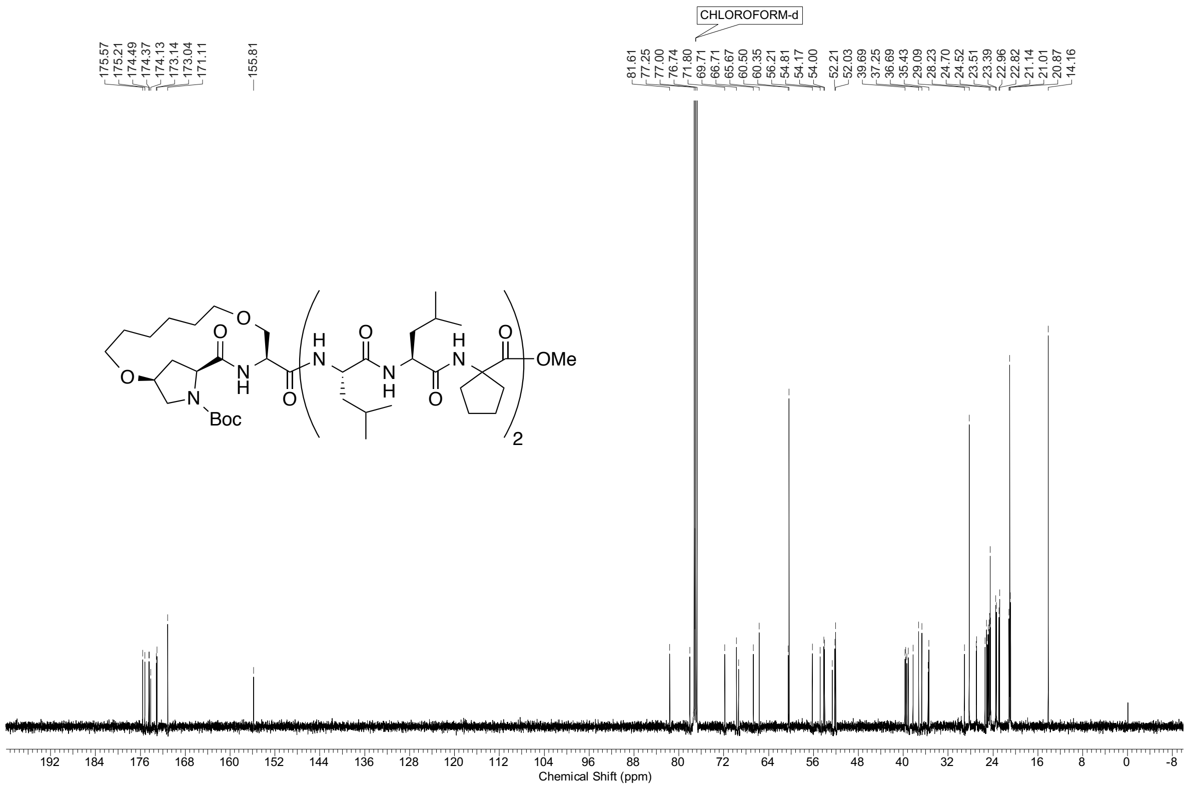

^{1}H NMR of **C'** (500 MHz, $CDCl_3$)

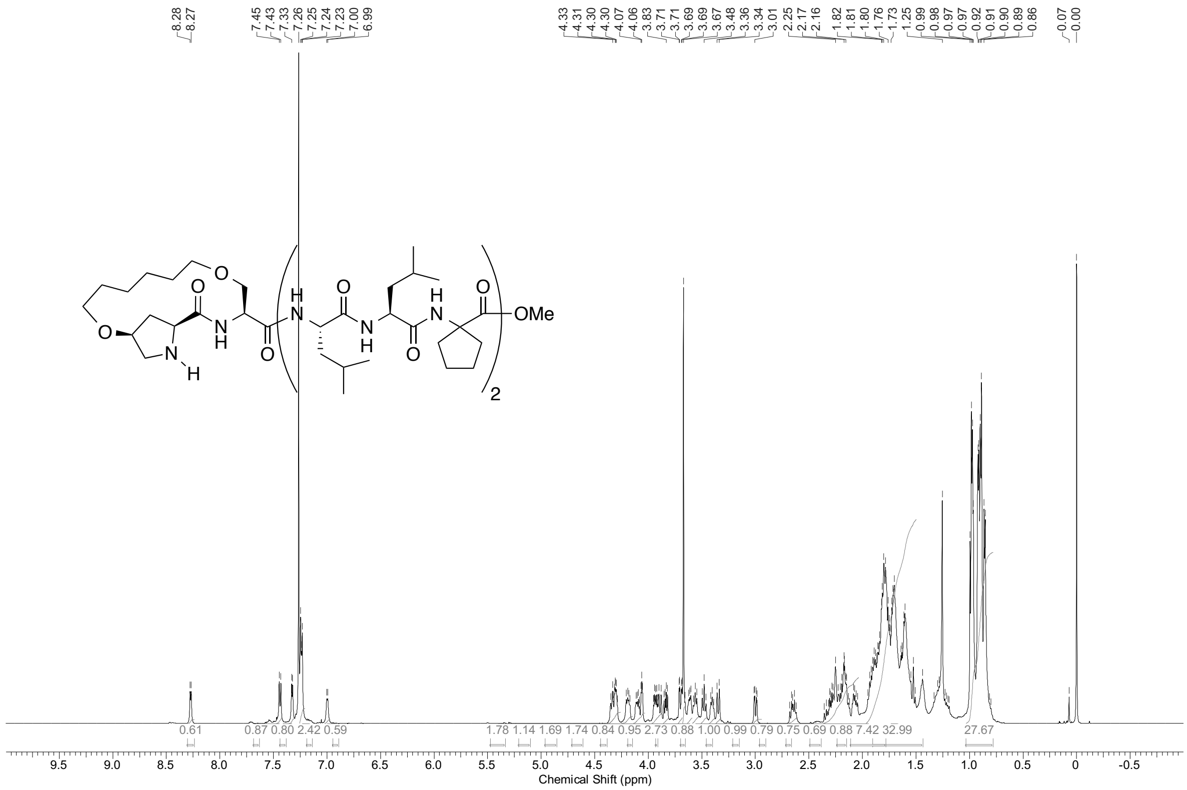

^{13}C NMR of **C'** (125 MHz, $CDCl_3$)

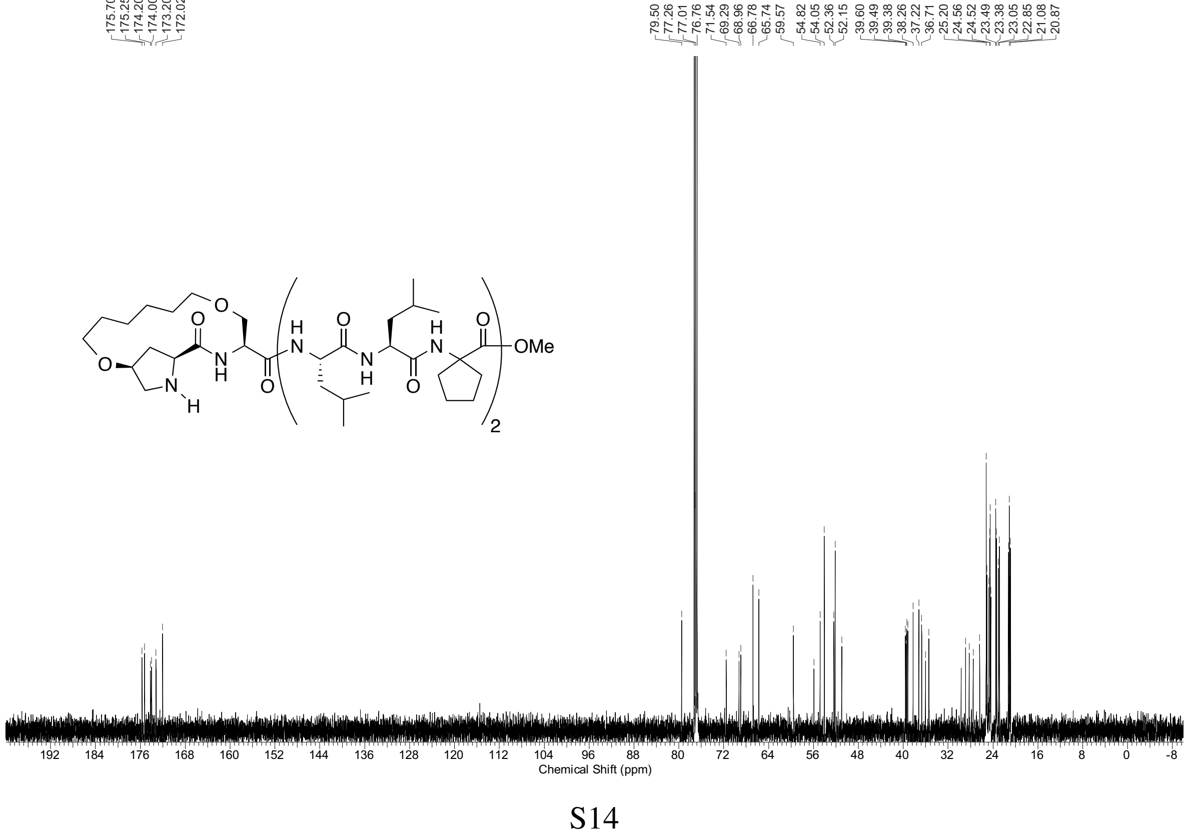

^{1}H NMR of **14** (500 MHz, $CDCl_3$)

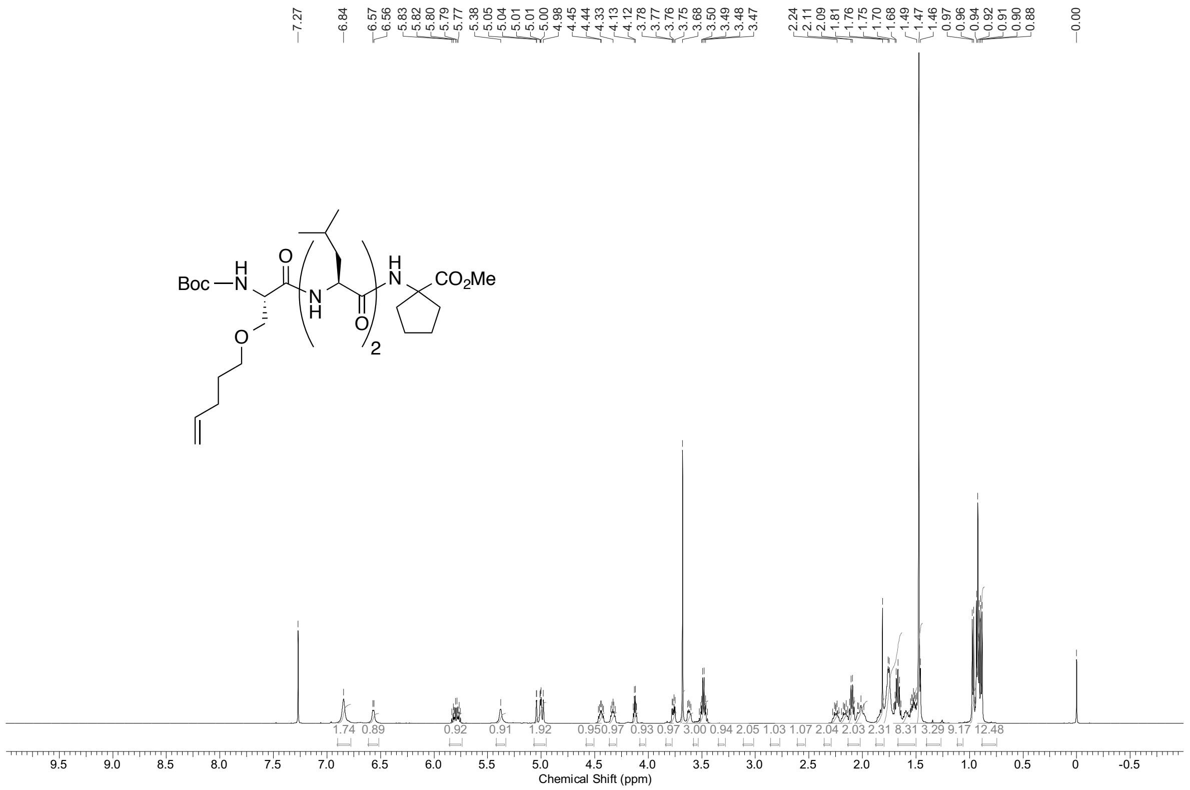

^{13}C NMR of **14** (125 MHz, $CDCl_3$)

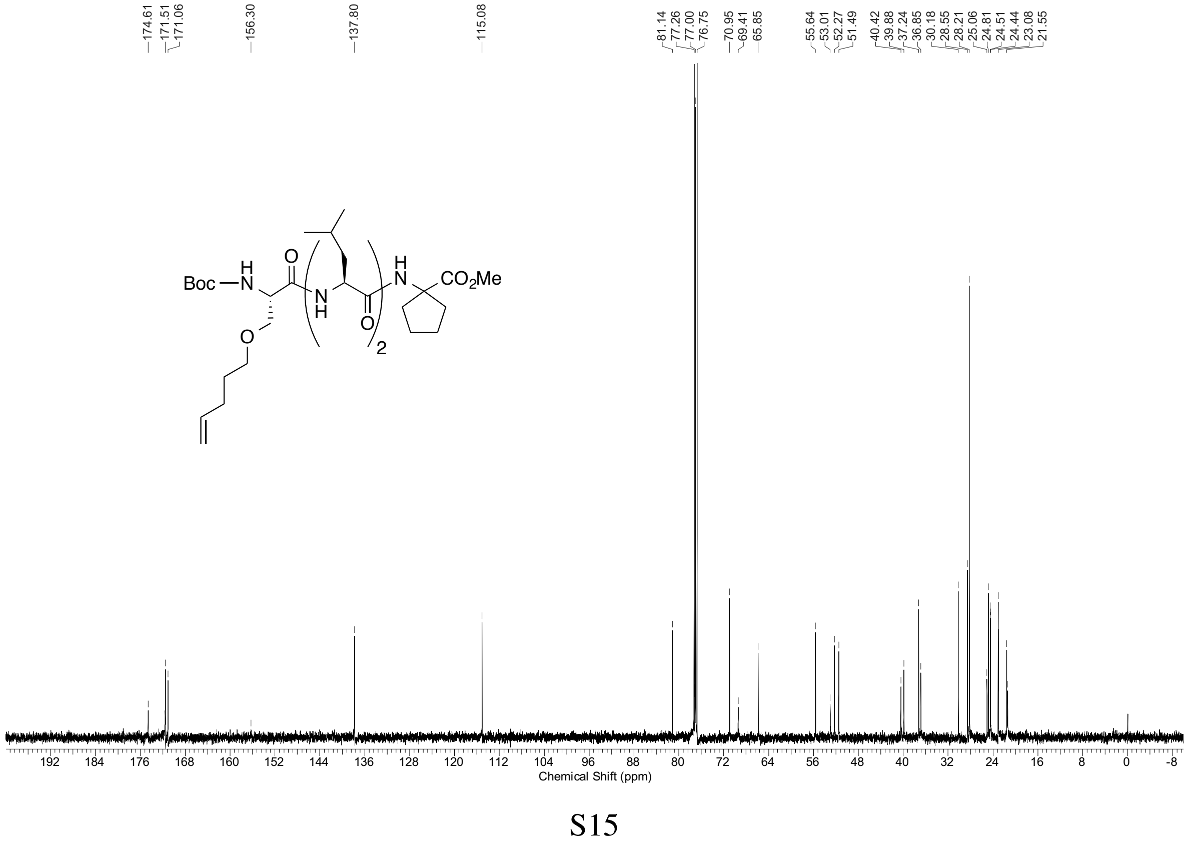

^{1}H NMR of **16** (500 MHz, $CDCl_3$)

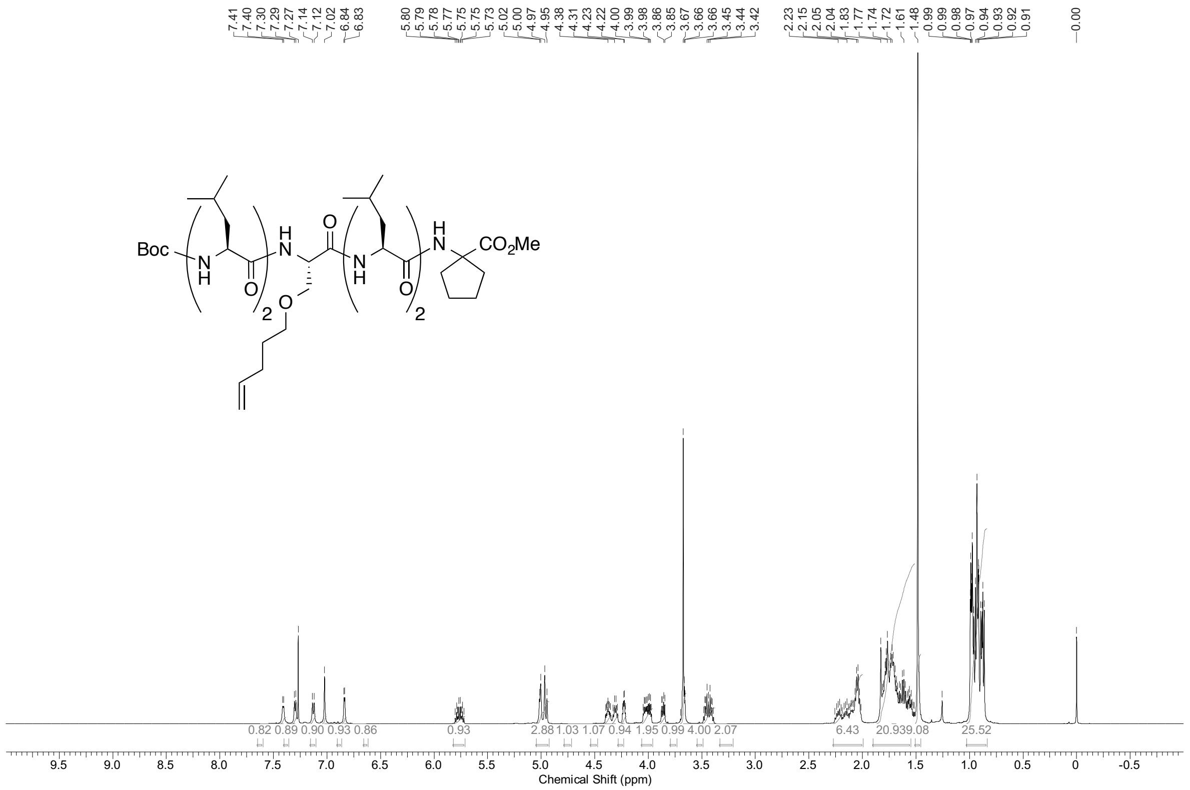

^{13}C NMR of **16** (125 MHz, $CDCl_3$)

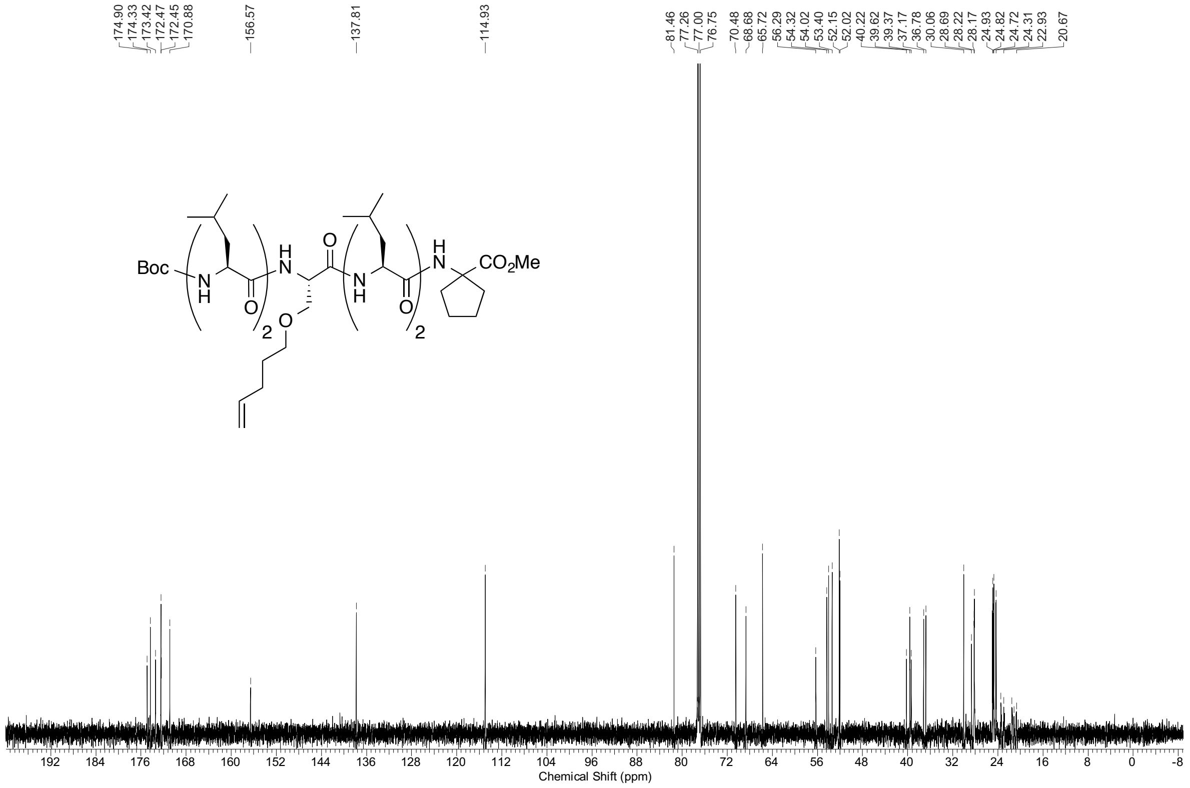

^{1}H NMR of **19** (500 MHz, $CDCl_3$)

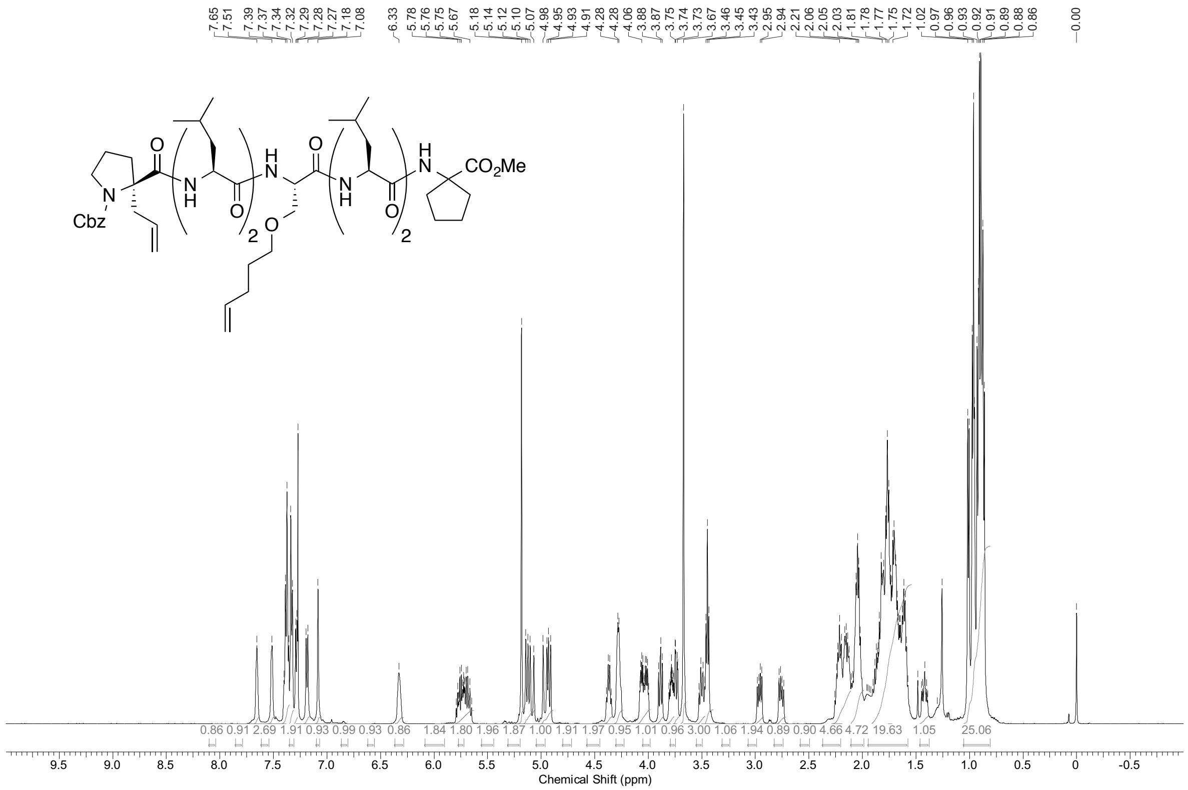

^{13}C NMR of **19** (125 MHz, $CDCl_3$)

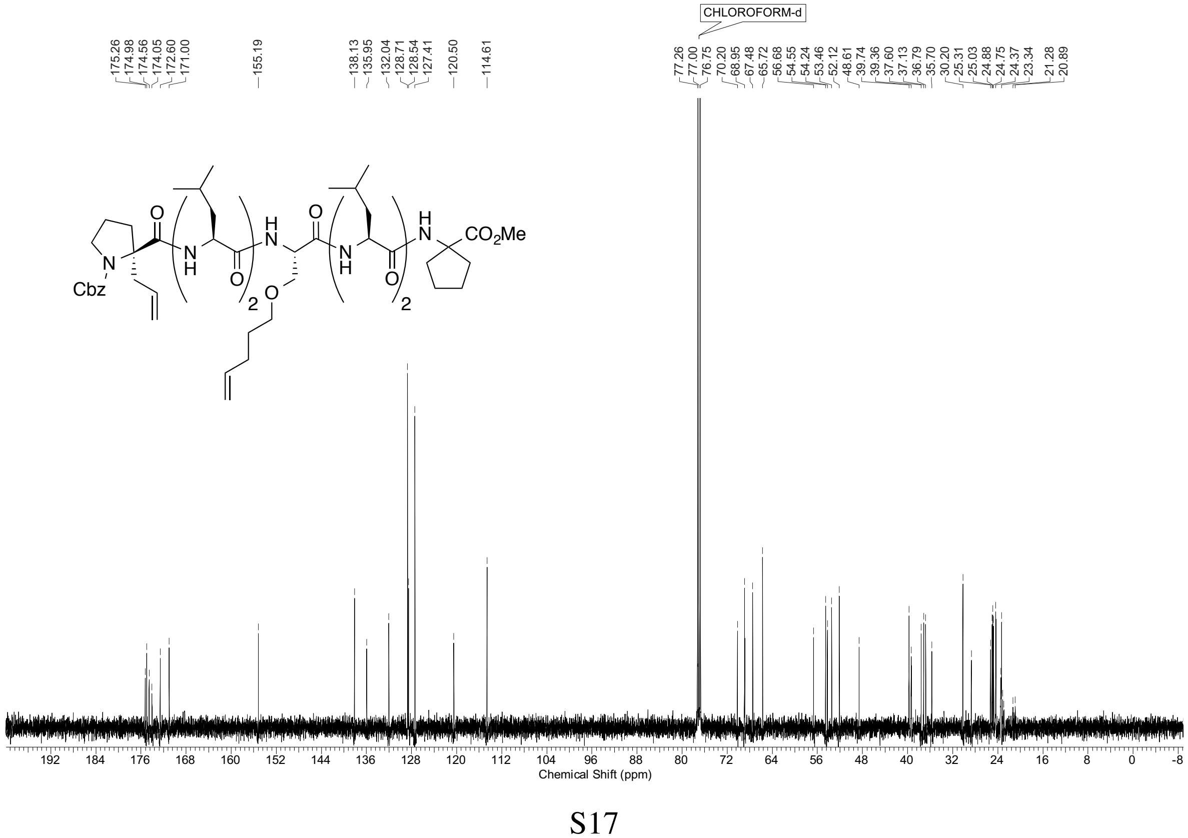

^{1}H NMR of **D** (500 MHz, $CDCl_3$)

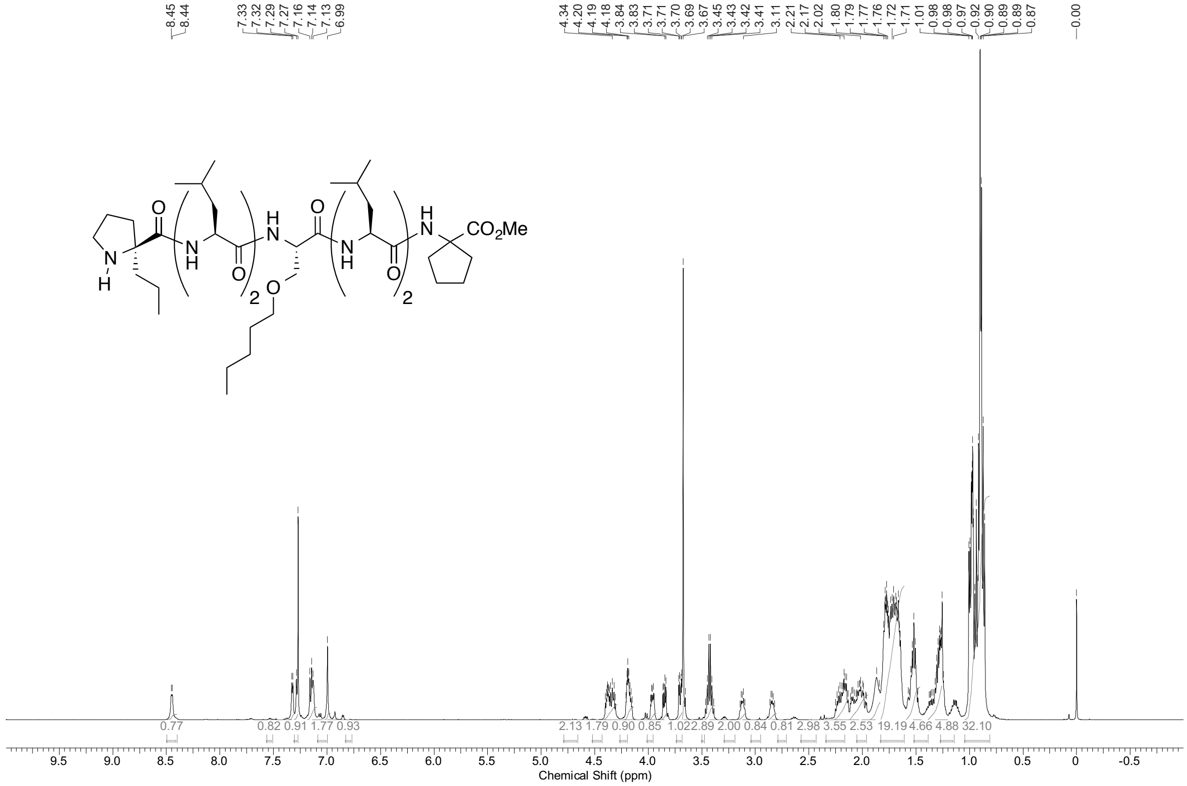

^{13}C NMR of **D** (125 MHz, $CDCl_3$)

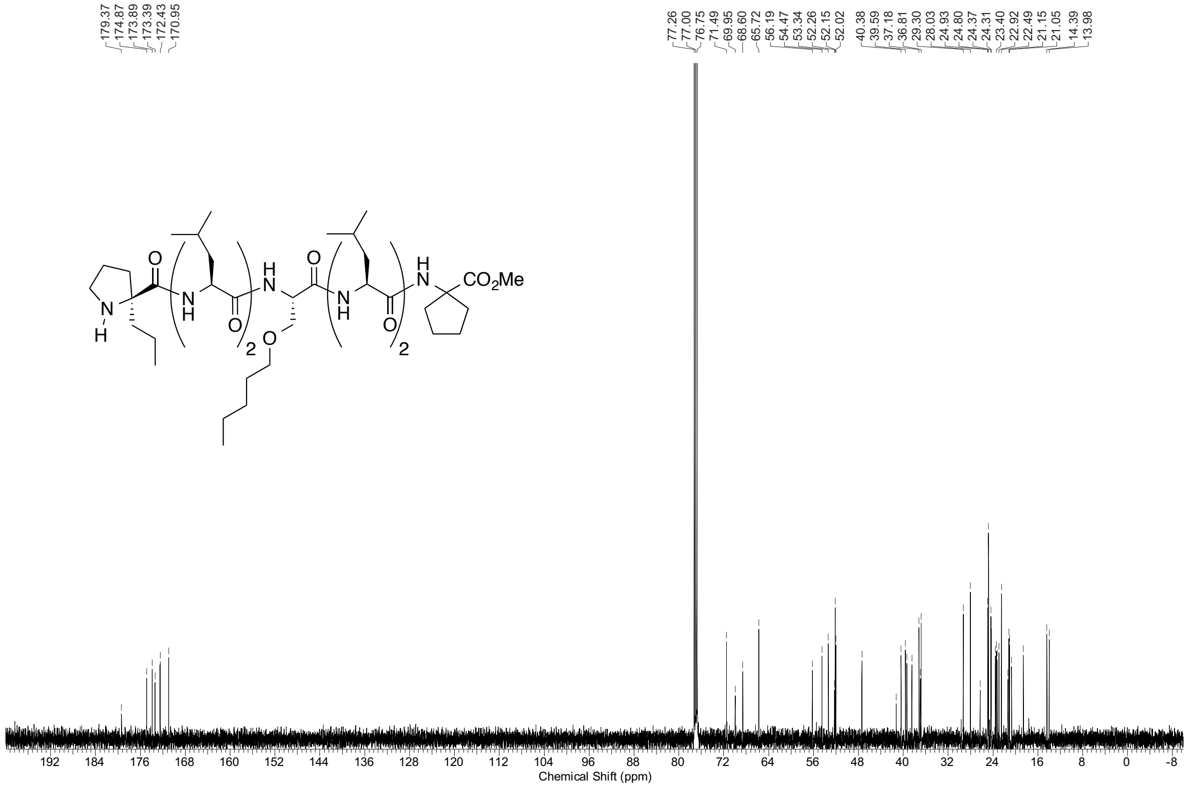

^1H NMR of **D’** (500 MHz, $CDCl_3$)

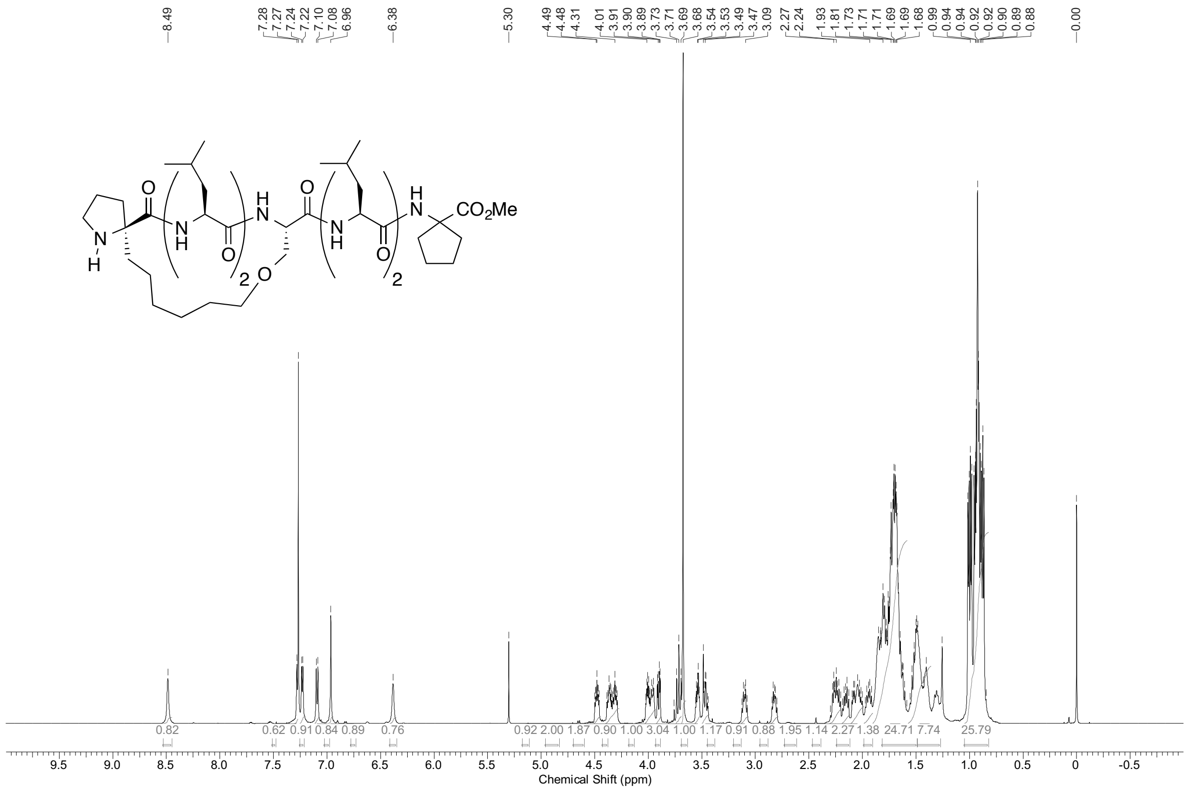

^{13}C NMR of **D’** (125 MHz, $CDCl_3$)

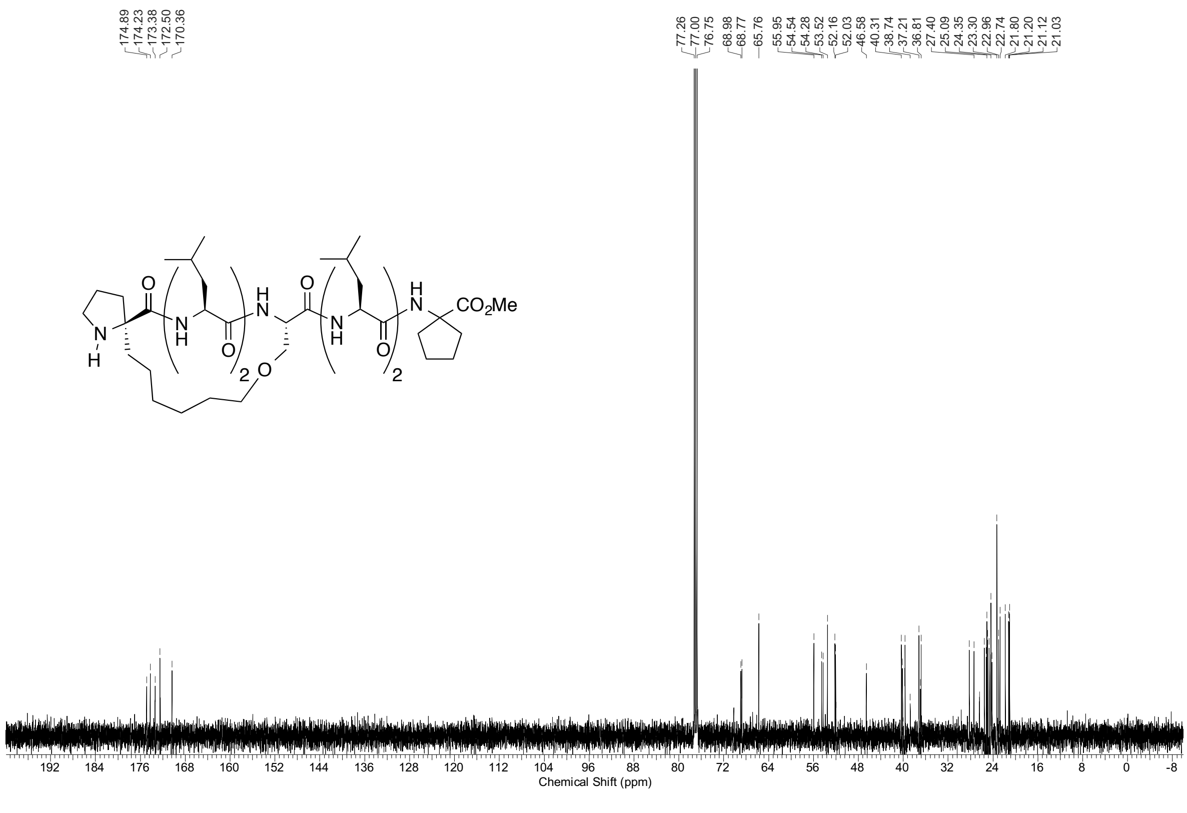

2. HPLC chart of compound 23.

Condition: Chiralpak AD-H, 10% *i*-propanol/*n*-hexane, flow rate 1.0 mL/min.

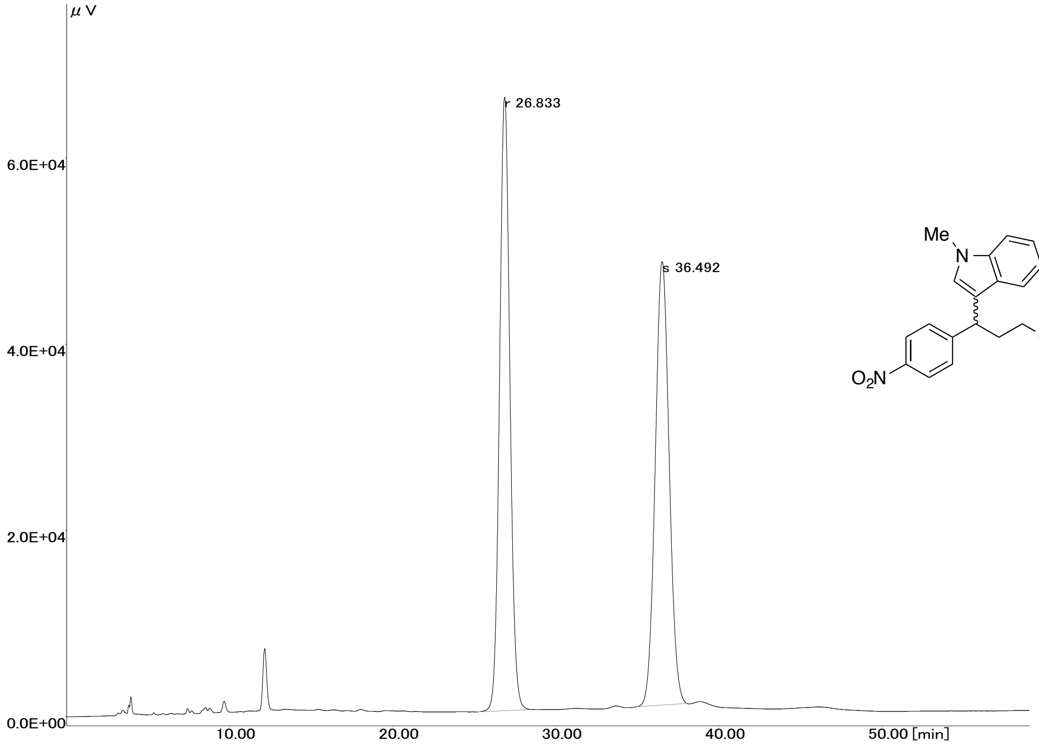

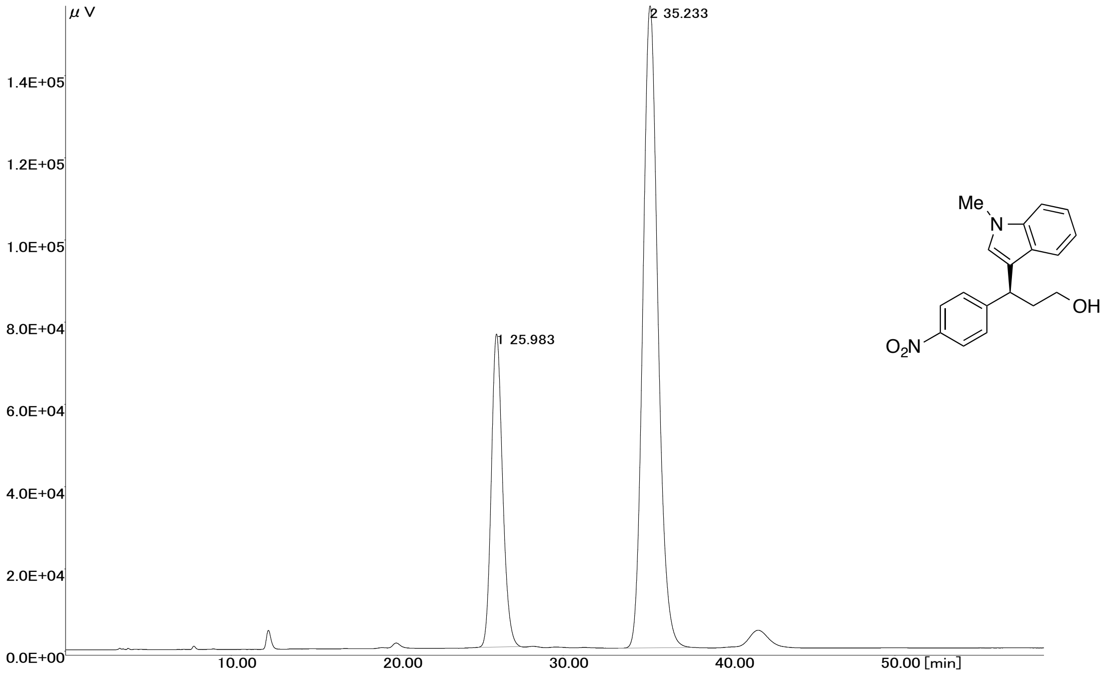

	Retention time (min)	Area	Area (%)
1	25.983	3530043	26.62
2	35.233	9732204	73.38
Total		13262247	100
